# A Universal Strategy of Anti‐Tumor mRNA Vaccine by Harnessing “Off‐the‐Shelf” Immunity

**DOI:** 10.1002/advs.202401287

**Published:** 2025-01-06

**Authors:** Jiayan Fu, Shuangqi Wu, Nengcheng Bao, Lili Wu, Huiru Qu, Zhechao Wang, Haiyang Dong, Jian Wu, Yongfeng Jin

**Affiliations:** ^1^ National Key Laboratory of Advanced Drug Delivery and Release Systems Zhejiang University Hangzhou Zhejiang 310058 China; ^2^ MOE Laboratory of Biosystems Homeostasis & Protection Innovation Center for Cell Signaling Network College of Life Sciences Zhejiang University Hangzhou Zhejiang 310058 China; ^3^ Department of Hepatobiliary and Pancreatic Surgery The First Affiliated Hospital Zhejiang University School of Medicine Hangzhou Zhejiang 310006 China

**Keywords:** anti‐tumor, mRNA vaccine, proof of concept, spontaneous cancer regression, universal strategy

## Abstract

Personalized neoantigen cancer mRNA vaccines are promising candidates for precision medicine. However, the difficulty of identifying neoantigens heavily hinders their broad applicability. This study developed a universal strategy of anti‐tumor mRNA vaccine by harnessing “off‐the‐shelf” immunity to known antigens. First, the model antigen ovalbumin (OVA) is used for mRNA vaccine design. In vitro test indicated that this mRNA vaccine reprogrammed tumor cells that can be recognized and killed by OVA‐specific cytotoxic T lymphocytes (CTLs). In situ mRNA vaccine notably inhibited tumor growth across three subcutaneous solid tumor models in mice. Further single‐cell sequencing analyses revealed that mRNA vaccination act to reshape the immunosuppressive tumor microenvironment (TME) toward more proinflammatory characteristics. Strikingly, this framework of mRNA‐based strategy can be applied to two clinical pathogen antigens, hepatitis B surface antigen (HBsAg), and SARS‐CoV‐2 spike receptor‐binding domain (SRBD). Interestingly, the mRNA‐based strategy largely recapitulated the scenario of spontaneous cancer regression following pathogen infection or vaccination. Collectively, this study provides not only proof of concept for universal anti‐tumor mRNA therapy, but also mechanistic insights in echoing the long‐standing puzzle of spontaneous cancer regression.

## Graphical abstract

This study developed a universal strategy of anti‐tumor mRNA vaccine by harnessing “off‐the‐shelf” immune memory to known antigens (OVA, HBsAg, and SRBD). These mRNA vaccines reprogrammed tumor cells and redirected antigen‐specific memory T cells to the lesion. In situ mRNA vaccination notably inhibited tumor growth across three solid tumor models. Further analyses revealed that it reshaped the immunosuppressive tumor microenvironment.

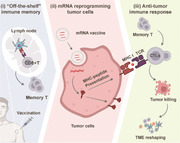

## 1 Introduction

Therapeutic mRNA cancer vaccines hold great promise for clinical cancer immunotherapy. Personalized neoantigen vaccines are now considered as candidates for precision medicine.^[^ ^1^ ^]^ Recently, the development of mRNA technology has facilitated the rapid delivery of individualized neoantigen vaccines, with the advantages of advanced security, efficiency, and bulk industrial manufacturing within a short time.^[^ ^2^ ^]^ However, the difficulty in identifying neoepitopes heavily hinders the broad applicability of personalized vaccines. More disturbingly, these techniques also require time and resources inherent for optimal validation of immunogenic neoepitopes and subsequent vaccine production.^[^ ^3^ ^]^ Distinguished from personalized vaccines, universal vaccines typically encode shared antigens that are expressed in a larger subset of patients or tumor types.^[^ ^4^ ^]^ Furthermore, they can be assessed and produced with standardized procedures, thus allowing wider applicability.^[^ ^5^ ^]^ Universal vaccines have been the primary focus of preclinical and clinical research.^[^ ^6^ ^]^ Universal cancer vaccines traditionally target tumor‐associated antigens (TAAs), which can be further divided into development‐specific (e.g., WT1), cancer‐testis (e.g., NY‐ESO‐1), tissue‐specific (e.g., gp100), or tumor‐overexpressed (e.g., P53) antigens.^[^ ^7^ ^]^ However, the therapeutic effects of traditional universal mRNA cancer vaccines are still constrained due to cancer antigenic heterogeneity and insufficient immunogenicity.^[^ ^6^ ^]^

The spontaneous regression (SR) of cancer, which is a rare but well‐documented phenomenon, possibly provide some clues for the development of universal cancer vaccines. Previous anecdotal reports have indicated complete SR following acute pathogen infection,^[^ ^8^ ^]^ dating back to 1891 when Coley first used *Streptococcus pyogenes* for the treatment of patients. With the recent COVID‐19 pandemic, there have been increasing reports of SR even in the absence of specific cancer treatment.^[^ ^9^ ^]^ For example, cases of regression of both Hodgkin lymphoma and follicular lymphoma have been reported following SARS‐CoV‐2 infection.^[^ ^10^ ^]^ Based on these observations, many studies investigated the mechanisms underlying the anti‐tumor effects, including cross‐priming, overexuberant immune responses, or oncolytic properties.^[^ ^11^ ^]^ Notably, SR occurred in some patients who had not been infected but had been vaccinated,^[^ ^9^, ^12^ ^]^ suggesting the intriguing possibility that SR of cancer may be due to the activation of immune memory generated by pathogen infection or vaccination.

Immune memory leads to specific protection against an antigen or target pathogen by generating long‐lived T cells. Upon antigen reexposure, these memory T cells execute “sensing and alarm” functions with high vigilance and cytotoxicity.^[^ ^13^ ^]^ Previous strategies have recapitulated several reinfection events for local cancer immunotherapy.^[^ ^14^ ^]^ The majority of these strategies have utilized virus infection to achieve immunity and delivered peptides derived from viral epitopes for triggering the pre‐existing T cell response,^[^ ^14^, ^15^ ^]^ what we refer to as “off‐the‐shelf” immunity. However, the delivered peptide epitopes were typically limited to 8–11 amino acid residues in length. By contrast, mRNA‐based therapy allows the application of full‐length antigen with higher safety, broader cross‐presentation, and less MHC allele restriction. As the COVID‐19 pandemic has irreversibly conferred immunity to viral epitopes upon a significant proportion of the world's population, it's worth evaluating whether mRNA vaccines could be utilized for anti‐tumor therapy by harnessing this type of “off‐the‐shelf” immunity.

In this study, using mRNA technology, we developed a universal anti‐tumor strategy by harnessing “off‐the‐shelf” immunity to known antigens. Unlike traditional shared vaccine, the mRNA vaccines used here do not encode intrinsic tumor antigens, but instead, serve as “labels” for in situ tumor reprogramming. We first used the model antigen ovalbumin and in vitro testing indicated that this mRNA vaccine reprogrammed tumor cells, enhancing CTL‐mediated killing. Strikingly, the in situ mRNA vaccine strongly inhibited tumor growth across three subcutaneous solid tumor models: melanoma B16‐F10, triple‐negative breast cancer 4T1, and colonic carcinoma CT‐26. The combination of bulk RNA‐seq and single cell RNA‐seq (scRNA‐seq) analyses revealed that mRNA vaccination reshaped the immunosuppressive TME toward more proinflammatory characteristics, thereby turning cold tumors into hot ones. Additionally, this strategy was applied to two clinical antigens: hepatitis B surface antigen (HBsAg) and SARS‐CoV‐2 spike receptor‐binding domain (SRBD). Collectively, this study provides proof of concept for a universal strategy of anti‐tumor mRNA therapy. Since our mRNA‐based strategy largely recapitulates the naturally occurring scenario of spontaneous cancer regression following pathogen infection or vaccination, our study improved our understanding of the mechanisms of spontaneous cancer regression.

## 2 Results

### 2.1 Characterization of mRNA Vaccine Using Model Antigen Ovalbumin

**Figure 1:**
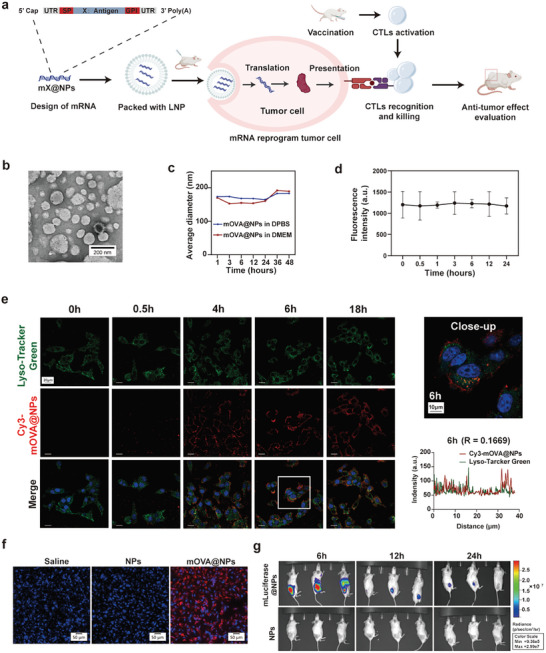
Characterization of mRNA vaccine using model antigen ovalbumin. a) Schematic of the universal strategy of anti‐tumor mRNA vaccine. b) Morphology of mOVA@NPs imaged by transmission electron microscope. Scale bar, 200 nm. c) The size of mOVA@NPs in DPBS or medium during evaluation over 48 h. d) Evaluation of the ability of the NPs to protect mRNA from degradation by RNase. a.u., arbitrary units. e) Cellular uptake and endo/lysosomal escape of Cy3‐mOVA@NPs characterized at different time points in B16‐F10 cells. Cy3‐mOVA@NPs (red), Endo/Lysosome (Lyso‐Tracker Green), DAPI (blue). Scale bars, 20 µm and 10 µm in high‐power view. Quantification of colocalization at 6 h. R, Pearson's correlation coefficient. f) Microscopic imaging of mcherry‐tagged OVA (red) expression in B16‐F10 cells transfected with saline, NPs, and mOVA@NPs for 48 h. Scale bars, 50 µm. g) Transfection of mRNA in vivo. Balb/c mice received intratumoral administration of mluciferase@NPs or LNP solvent alone as a control (20 µg mRNA per mouse) (n = 3). Bioluminescence was measured at 6, 12, and 24 h using an in vivo imaging system. The positions of three mice were assigned randomly during imaging.

Upon antigen reexposure, memory T cells generated by vaccination execute functions with high vigilance and cytotoxicity.^[^ ^13^ ^]^ To achieve the loal activation of these pre‐existing T cell responses, we designed a universal strategy of anti‐tumor mRNA vaccine. We hypothesized that the intratumoral delivery of the mRNA vaccine may reprogram tumor cells to present universal antigen epitopes on their surfaces. This reprogramming would thereby redirect antigen‐specific memory T cells to the tumor site, thus enhancing anti‐tumor responses and promoting antigen spread. For antigen selection in this vaccine, we used one model antigen (OVA) and two clinical antigens (HBsAg and SRBD) (**Figure** **1**a). First, synthesis of OVA mRNA was followed by its encapsulation into the core of lipid nanoparticles (LNPs) (Figure S1a, Supporting Information), designated as mOVA@NPs. As the antigens are interchangeable, this type of mRNA vaccine is designated as mX@NPs; E.g., when HBsAg, the formulation is designated as mHBsAg@NPs.

We first characterized the physicochemical properties of this vaccine formulation. Transmission electron microscopy (TEM) and agarose gel electrophoresis indicated a spherical morphology with satisfactory nanometer‐scale size and stability (Figure 1b‐d; Figure S1b‐d, Supporting Information). In the cell line B16‐F10, the intracellular red fluorescence from Cy3‐mOVA@NPs increased with prolongation of incubation time (Figure 1e; Figure S1e, Supporting Information). Most of the red fluorescence was not colocalized with the green fluorescence from lysosomes at 6 h (Figure 1e; Figure S1f, Supporting Information), indicating effective endosomal escape of the mOVA@NPs into the cytoplasm. Next, to evaluate the in vitro translation efficacy of mOVA@NPs, we fused OVA with a mcherry tag. Confocal laser scanning microscopy (CLSM) combined with flow cytometry revealed high translation efficiency with modest cytotoxicity (Figure 1f; Figure S1g,h, Supporting Information). As expected, the translated products were tethered outside the cell membrane (Figure S1i‐k, Supporting Information) because a C‐terminal glycosylphosphatidylinositol anchor was fused to the antigen.^[^ ^16^ ^]^ Additionally, when OVA antigen was substituted with luciferase, intratumoral administration of mluciferase@NPs exhibited an obvious fluorescent signal (Figure 1g), confirming the feasibility of intratumoral delivery of the mRNA vaccine. Taken together, these results indicate that this mRNA vaccine formulation is pharmacologically valid, justifying further investigation.

### 2.2 mOVA@NPs Reprogramed Tumor Cells that Could be Recognized and Killed by OVA‐Specific CTLs

**Figure 2:**
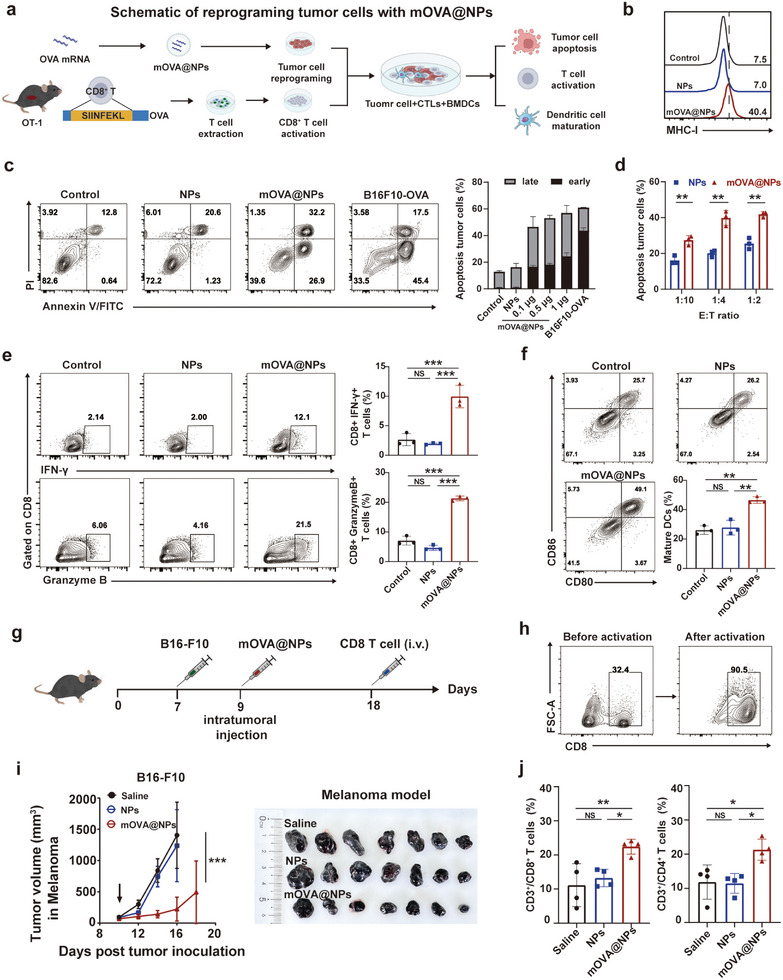
mOVA@NPs reprogrammed tumor cells that could be recognized and killed by OVA‐specific CTLs. a) Schematic of reprograming tumor cells with mOVA@NPs to investigate the killing efficiency of OVA‐specific CTLs and maturation of BMDCs. b) Flow cytometric analysis of the MHC‐I level in tumor cells 12 h after transfection with mOVA@NPs. c) Flow cytometric analysis and quantification of the level of apoptosis in B16‐F10 cells 12 h after transfection with different dosages of mOVA@NPs or B16F10‐OVA cells as a positive control (*n*  =  3). d) In vitro cytotoxicity assays performed with activated CD8^+^ T cells and B16‐F10 cells pretreated with mOVA@NPs (*n*  =  3). Two‐tailed unpaired Student's *t* test. e) Flow cytometry‐based quantification of IFN‐γ^+^CD8^+^ T cells and granzyme B^+^ CD8^+^ T cells after coculture with mOVA@NPs for 12 h (*n*  =  3). f) Flow cytometric analysis of BMDC maturation biomarkers (CD80 and CD86) (*n*  =  3). g) Experimental timeframe for administration of mOVA@NPs and CD8^+^ T cell venous reinfusion in B16‐F10 tumor‐bearing mice. h) Flow cytometric analysis of the activation of CD8^+^ splenocyte cells of OT‐1 mice. i) The average tumor volume curves and tumor image of the mouse model of melanoma (*n*  =  7). j) Flow cytometric analysis of the levels of CD3^+^/CD8^+^and CD3^+^/CD4^+^ T cells in tumor tissues (*n*  =  4). All data are presented as the mean ± SD. One‐way ANOVA with the Tukey's multiple comparisons test was used for all statistical analyses. *P < 0.05; **P < 0.01; ***P < 0.001; NS, not significant.

Antigen‐specific immune recognition and eradication of infected cells require CTLs, which ensure a swifter response upon antigen reexposure.^[^ ^17^ ^]^ To explore whether mOVA@NPs could reprogram tumor cells to prime OVA‐specific CTLs for cellular killing, we used OT‐1 mice to investigate the killing efficiency of CD8^+^ T cells against OVA peptides on the tumor cell surface. T cell receptors from OT‐1 mice containing transgenic inserts for Tcra‐V2 and Tcrb‐V5 genes were designed to specifically recognize OVA residues_257‐264_ (SIINFEKL) (**Figure** **2**a). Notably, the expression level of MHC‐I in tumor cells transfected with mOVA@NPs was highly elevated (Figure 2b). Next, to validate the cell killing efficiency in vitro, activated cytotoxic CD8^+^ T cells isolated from OT‐1 mice were cocultured with B16‐F10 tumor cells transfected with mOVA@NPs. The coculture resulted in a significant increase in the rate of apoptotic tumor cells (Figure 2c). Approximately 50% of the B16‐F10 cells underwent apoptosis at an effector‐to‐target (E: T) ratio of 5:1 and the rate of apoptosis increased with higher doses of mOVA@NPs (Figure 2c). Even at low E:T ratios, this reprogramming still resulted in some degree of target cell killing (Figure 2d). Additionally, the proportion of CTLs that secreted interferon‐gamma (IFN‐γ) and granzyme B significantly increased after coculturing with reprogrammed tumor cells (Figure 2e). These observations indicate that reprogrammed tumor cells were killed by CTLs through the release of cytotoxic molecules.

To further investigate the immune stimulation of dendritic cells (DCs) induced by CTL‐mediated cell killing, B16‐F10 cells were treated with PBS (control), blank NPs, or mOVA@NPs, followed by coculture with OVA‐specific CTLs and bone marrow‐derived dendritic cells (BMDCs). As shown in Figure 2f, reprogrammed target cells with mOVA@NPs resulted in significant upregulation of CD80 and CD86 surface expression on BMDCs by 1.8‐fold compared to the control group. Taken together, these in vitro assays indicate that mOVA@NPs reprogramed tumor cells that could be recognized and killed by CTLs, which efficiently induced the maturation of DCs via CTL‐mediated cell killing.

To simulate a more realistic scenario, we conducted similar reprogramming experiments in subcutaneous B16‐F10‐bearing mice in vivo. Primary splenocytes from OT‐1 mice were extracted and activated with OVA_257‐274_ peptide and IL‐2. Flow cytometry analysis demonstrated that adoptively transferred effector T cells were efficiently recruited to the neoplastic lesion, leading to strong inhibition of tumor growth (Figure 2g‐j). Taken together, these results indicate that mOVA@NPs reprogramed tumors both in vivo and in vitro, which could then be recognized and killed by OVA‐specific CTLs.

### 2.3 mOVA@NPs Elicit Strong Anti‐Tumor Effects in Three Cancer Models

**Figure 3:**
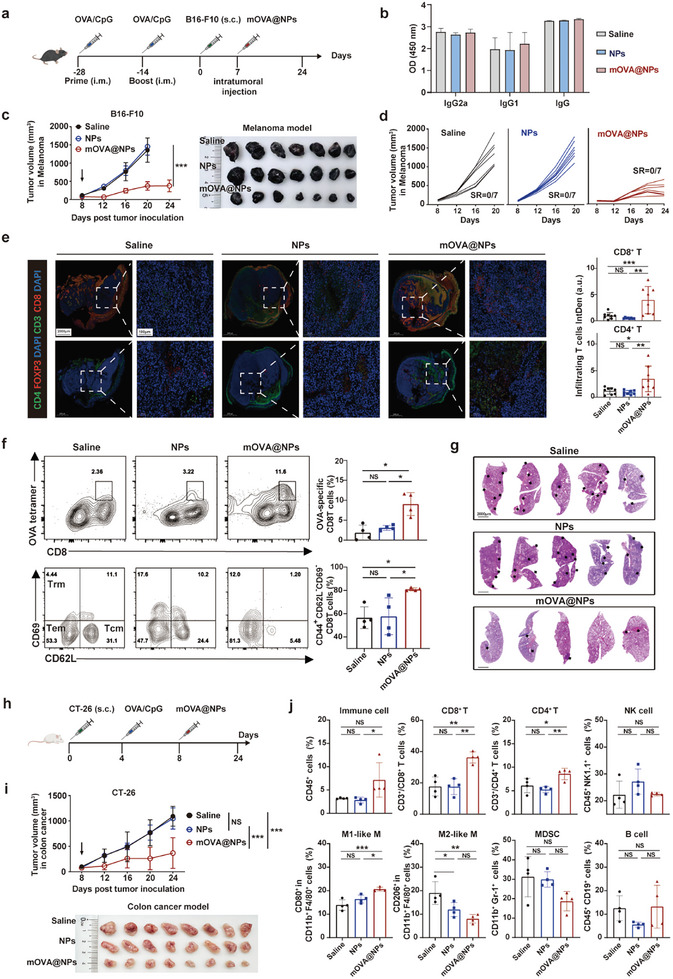
mOVA@NPs elicited strong anti‐tumor effects in three cancer models. a) Experimental timeframe for OVA vaccination and administration of mOVA@NPs in B16‐F10 tumor‐bearing mice. b) OVA‐specific IgG1, IgG2a, and IgG antibody titers determined by ELISA in the melanoma model (*n*  =  7). c) Average tumor volume curves and tumor images of a mouse model of melanoma (*n*  =  7). d) Individual tumor volume curves of mouse model of melanoma (*n*  =  7). e) Immunostaining of CD3, CD8, CD4, and FOXP3 was performed and the signal intensity was quantified using ImageJ. Representative images of indicated staining of tumor tissues are shown. Scale bars, 2000 µm (low magnification) and 100 µm (high magnification). f) Flow cytometric analysis of OVA tetramer^+^CD8^+^ T cells (upper panel) and expression levels of CD69 and CD62L gated on CD44^+^CD8^+^ T cells (lower panel) (*n*  =  4). g) Hematoxylin and eosin (H&E) staining of lung metastases in mouse model of breast cancer. Scale bars, 2000 µm. h) Experimental timeframe for OVA vaccination and administration of mOVA@NPs in CT‐26 tumor‐bearing mice. i) Average tumor volume curves and tumor images of mouse model of colon cancer (*n*  =  8). j) Flow cytometric quantification of immune cells within CT‐26 tumors. The proportions of CD45^+^ cells, CD3^+^/CD8^+^ T cells, CD3^+^/CD4^+^ T cells, NK cells, M1‐TAM, M2‐TAM, MDSC, and B cells are shown. All data are presented as the mean ± SD. One‐way ANOVA with the Tukey's multiple comparisons test was used for statistical analyses. *P < 0.05; **P < 0.01; ***P < 0.001; NS, not significant.

We next investigated the anti‐tumor effects of this mOVA@NPs mRNA vaccine in vivo. The “prime‐and‐boost” vaccination schedule was shown to generate “off‐the‐shelf” immunity (**Figure** **3**a). Both groups exhibited strong anti‐OVA IgG responses, and the proportion of the serum IgG2a of total IgG was higher than that of IgG1 (Figure 3b), indicating that the differentiation of OVA‐specific T cells trended toward the Th1 response. In this case, only tumor‐bearing mice harbored pre‐existing OVA‐specific immunity that significantly suppressed tumor growth (Figure S2a‐d, Supporting Information). Additionally, when LNPs loaded with nonsense mRNA (mNS@NPs) were injected intratumorally into mice, no significant differences in tumor suppression were observed (Figure S2a, Supporting Information), indicating that the anti‐tumor efficacy was not dominated by innate immune activation by mRNA itself. Both mOVA@NPs and NPs were well tolerated by the mice at the administration dose, with almost no loss of body weight (Figure S2b, Supporting Information). Notably, tumor proliferation was inhibited by 75.9% in the mOVA@NPs‐treated group (Figure 3c,d). Immunofluorescence staining of tumors showed that mOVA@NPs significantly enhanced the frequency of tumor‐infiltrating effector T cells and helper T cells (Figure 3e), but slightly reduced the level of regulatory T cells (Treg) to some extent (Figure S2e, Supporting Information). To further evaluate the activation of tumor‐infiltrating effector T cells, MHC‐I tetramer staining analysis gated on CD8 T cells confirmed that local administration of mOVA@NPs effectively redirected antigen‐specific effector T cells to the lesion (Figure 3f, upper panel). Moreover, we assessed whether this mRNA‐based strategy activated memory T cells. Indeed, tumors treated with mOVA@NPs exhibited a higher infiltration of CD44^+^CD8^+^ T cells compared to the control group (Figure S2f, Supporting Information). A substantial proportion of the tumor‐infiltrating memory T cells differentiated from central memory T cells (CD44^+^CD62L^+^CD69^−^) to effector memory T cells (CD44^+^CD62L^−^CD69^−^) (Figure 3f, lower panel; Figure S2g, Supporting Information). These observations implied a functional shift of local memory T cells from lymphoid homing to cytotoxic effector activity. A similar tendency of tumor growth suppression was observed in triple‐negative breast cancer 4T1 model (65.5%) (Figure S3a‐d, Supporting Information). HE staining showed a significant reduction in pulmonary metastases in mOVA@NPs‐treated mice (Figure 3g; Figure S3e,f, Supporting Information). Taken together, all these results indicate that mOVA@NPs elicit strong anti‐tumor effects in mouse models of melanoma and breast cancer.

Next, we examined another applicable scenario for this mRNA vaccine strategy in mice lacking pre‐existing OVA‐specific immunity. Specifically, we designed another set of vaccination timeframes in a colonic carcinoma CT‐26 model (Figure 3h). In contrast to the above models, OVA was vaccinated after tumor implantation to prime for “instant immunity”, and we observed tumor growth was visibly suppressed (66.42%) following local treatment with mOVA@NPs (Figure 3i). Consistently, we performed flow cytometric analysis to investigate changes in the immunosuppressive TME induced by this mRNA strategy. The data demonstrated that mOVA@NPs enhanced the recruitment of CD3^+^/CD8^+^ effector T cells and CD3^+^/CD4^+^ helper T cells to neoplastic lesions (Figure 3j). Additionally, tumor‐associated macrophages (TAMs) were modulated, showing an increased proportion of pro‐inflammatory M1‐TAMs and a decreased proportion of anti‐inflammatory M2‐TAMs, while small but insignificant decrease was observed for myeloid‐derived suppressor cells (MDSCs) (Figure 3j). These findings indicated that this strategy somehow reshaped the immunosuppressive TME. Collectively, all these results indicate that this mRNA vaccine strategy can elicit anti‐tumor immune responses across three cancer models, regardless of whether immunity is pre‐existing or just primed.

### 2.4 Bulk RNA‐Seq and scRNA‐Seq Revealed that mOVA@NPs Reshape the Immunosuppressive TME

**Figure 4:**
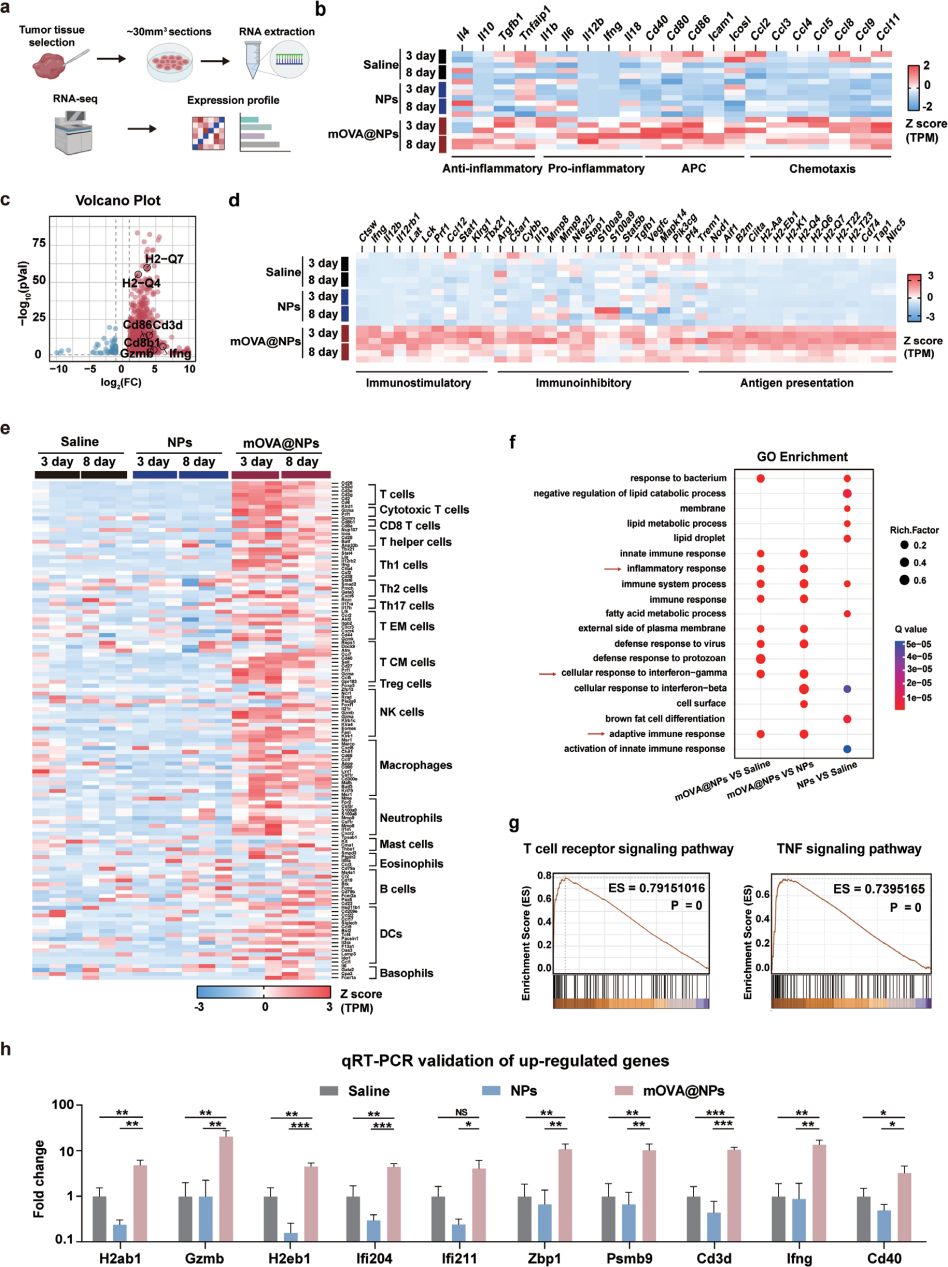
mOVA@NPs activate immunological processes and pathways at neoplastic lesion. a) Experimental design of RNA‐seq in C57BL/6 mice treated with saline, NPs, and mOVA@NPs for 3 and 8 days (*n*  =  3). b) Heat map of RNA‐seq data for anti‐inflammatory, pro‐inflammatory, APC, and chemotaxis‐related gene expression from harvested tumor tissues treated with saline, NPs, and mOVA@NPs, plotted as z scores of normalized gene expression for each gene. c) Volcano plot of RNA‐seq data for tumor tissues treated with NPs and mOVA@NPs. Differentially expressed genes were identified according to a threshold of |log_2_(fold change) | > 1 and false discovery rate (FDR) < 0.05. d) Heat map of the expression of immunomodulation genes from tumor tissues treated with saline, NPs, and mOVA@NPs. e) Heat map of expression of genes associated with immune cell types from tumor tissues treated with saline, NPs, and mOVA@NPs. f) Bubble plots of GO enrichment analysis. The top 10 GO terms with the lowest *P*‐values are shown. g) Enrichment scores of indicated pathways from tumor tissues treated with NPs and mOVA@NPs. h) Validation of significantly upregulated genes by RT‐qPCR (*n*  =  3). Data are presented as the mean ± SD. One‐way ANOVA with the Tukey's multiple comparisons test was used for all statistical analyses. *P < 0.05; **P < 0.01; ***P < 0.001; NS, not significant.

To elucidate the intrinsic mechanism underlying this mRNA strategy, we performed bulk RNA‐seq on B16‐F10 tumors after mOVA@NPs therapy for 3 and 8 days (**Figure** **4**a; Figure S4a, Supporting Information). In total, we identified 1261 differentially expressed genes (1183 upregulated and 78 downregulated) compared with the NPs control group (Figure S4b, Supporting Information). Notably, immunostimulatory and antigen presentation‐associated genes (MHC‐I class) were highly upregulated (Figure 4b‐d). Immune cell markers, especially for T cells (cytotoxic T cells, CD8 T cells, T helper cells), showed high enrichment in the mOVA@NPs group, suggesting an enhancement in the infiltration efficiency of T cells in the immunosuppressive TME (Figure 4e; Figure S4c, Supporting Information). Immunostimulatory and antigen presenting‐associated genes from tumors harvested on day 3 showed more upregulation than those from 8‐day tumors (Figure 4b‐e). Moreover, genes involved in multiple immune‐related GO pathways were highly upregulated, including those modulating the adaptive immune response and cellular response to IFN‐γ (Figure 4f; Figure S4f, Supporting Information). Notably, gene set enrichment analysis (GESA) revealed higher scores for T cell receptor signaling pathway, TNF signaling pathway, and antigen processing and presentation in the mOVA@NPs‐treated groups (Figure 4g; Figure S4d,e, Supporting Information). The findings of RNA‐seq were confirmed by RT‐qPCR (Figure 4h; Table S1, Supporting Information). Taken together, these results revealed that mOVA@NPs activate T cell‐mediated immunological processes and pathways by enhancing the presentation of endogenous antigens.

**Figure 5:**
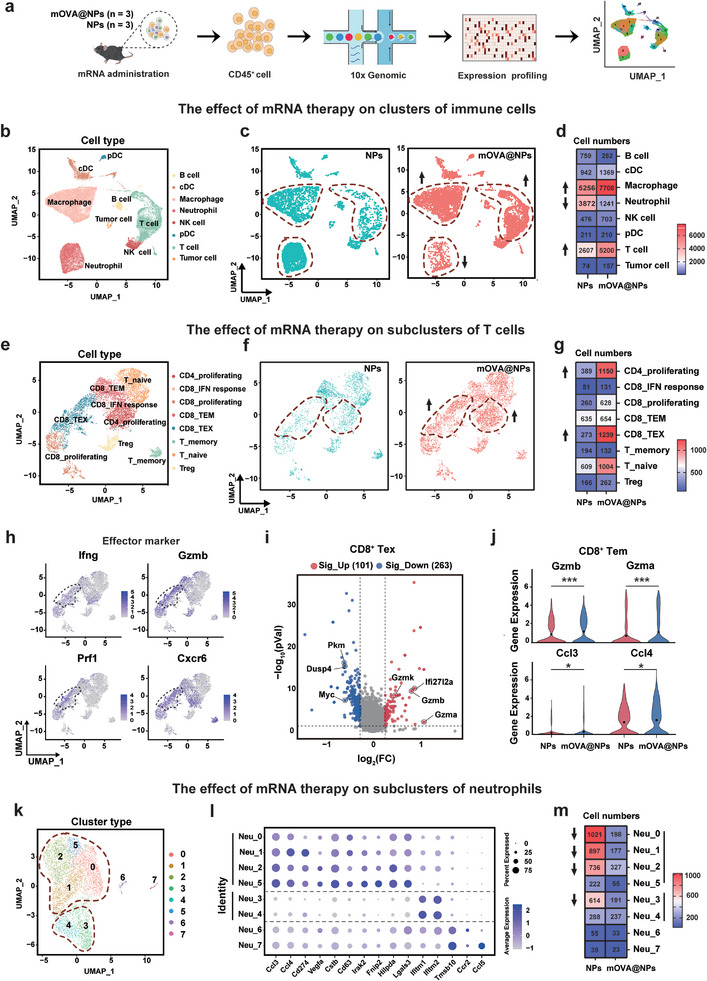
scRNA‐seq analyses revealed that mOVA@NPs reshape the immunosuppressive TME. a) Workflow of scRNA‐seq in C57BL/6 mice treated with NPs and mOVA@NPs for 3 days (*n*  =  3). b, c) UMAP plot of 31,067 immune cells isolated from melanoma tissues from mOVA@NP and NP groups, with each cell color coded for cell type (b) and sample group (c). d) Heat map showing the numbers of each immune cell subpopulation in the mOVA@NPs and NP groups. e, f) UMAP plot of annotated T cell subpopulations color coded by cell subpopulation (e) and sample group (f). g) Heat map showing the numbers of each T cell subpopulation in the mOVA@NPs and NP groups. h) UMAP plot showing the expression of effector markers genes (*Ifng*, *Gzmb*, *Prf1*, *Cxcr6*). i) Volcano plots of log_2_(fold change) and log_10_(adjusted *P*‐value) of differentially expressed genes in CD8^+^ Tex from NP‐treated tumors and mOVA@NP‐treated tumors. j) Violin plots of the expression levels of effector‐related genes in the CD8^+^ Tem subset in mOVA@NP‐treated tumors compared to the control NP group. Two‐tailed unpaired Student's t tests were used for statistical analyses. k) UMAP plot of neutrophil subpopulations color coded by cell subpopulation. l) Marker gene expression for each neutrophil subpopulation, with dot color and size representing the averaged scaled expression and percentage of marker gene expression, respectively. m) Heat map showing the numbers of each neutrophil subpopulation in the mOVA@NP and NP groups. *P < 0.05; **P < 0.01; ***P < 0.001; NS, not significant.

To further evaluate the impact of immune cells on the heterogeneous TME, immune cells (CD45^+^) were purified and subjected to scRNA‐seq (**Figure** **5**a). UMAP clustering revealed 23 subclusters with 8 major immune populations: T cells, B cells, NK cells, macrophages, neutrophils, conventional dendritic cells (cDCs), plasmacytoid dendritic cell (pDCs), and a small portion of sorted tumor cells (Figure 5b; Figure S5a‐d, Supporting Information). Of note, T cells and macrophages were enriched in mOVA@NPs‐treated tumors, whereas neutrophils and B cells showed higher infiltration in the NPs group (Figure 5c,d; Figure S5e, Supporting Information). To obtain deeper insights, further subgroup analyses of T cells identified eight subclusters, including naive T cells, memory T cells, Tregs, proliferating CD4^+^ T cells, IFN‐responsive CD8^+^ T cells, proliferating CD8^+^ T cells, effector memory CD8^+^ T cells (CD8^+^ Tem), and exhausted CD8^+^ T cells (CD8^+^ Tex) (Figure 5e; Figure S6a,b, Supporting Information). Notably, infiltrating CD4^+^ T cells and CD8^+^ T cells from mOVA@NPs‐treated tumors were highly enriched, which were largely assigned to the proliferating CD4^+^ T cells and the CD8^+^ Tex (Figure 5f). Remarkable distinction could be observed in both proportions and numbers of these two subpopulations between the mOVA@NPs and control groups (Figure 5g; Figure S6c, Supporting Information). Surprisingly, CD8^+^ Tex cells maintained an effector state with high expression of Ifng, Gzmb and Prf1 (Figure 5h). Differentially expressed genes (DEGs) analyses revealed that CD8^+^ Tex exhibited upregulation of effector and IFN‐stimulated genes (e.g., Gzmk, Gzmb, Gzma, Ifi27l2a) in the mOVA@NPs‐treated tumors (Figure 5i; Figure S6d,e, Supporting Information). Besides, we observed a functional shift of CD8^+^ Tem. This subcluster usually plays a crucial role since it rapidly responds to antigenic epitopes presented on the tumor surface.^[^ ^18^ ^]^ Genes associated with effector cytotoxicity (Gzma, Gzmb) and chemokines mediating cell migration and localization (Ccl3, Ccl4) were significantly upregulated (Figure 5j). This pattern likely reflects increased cytotoxic efficacy of CD8^+^ Tem. Furthermore, the expression levels of Tra and Trb (T cell receptor α and β chain) were significantly downregulated (Figure S6f, Supporting Information), implying a negative feedback regulation adapted to T cell activation following sustained antigen exposure.

In the macrophage subgroup, mRNA therapy led increases in both proinflammatory macrophage and Mrc macrophages, with a decrease in Vegfa macrophages in the TME (Figure S7a‐d, Supporting Information). Notably, Vegfa macrophages and Proinflammatory macrophages exhibited downregulation of tumor angiogenesis and protumorigenic genes (e.g., Cxcl3, Cxcl1, S100a8, SPP1) and upregulation of immune cell recruitment‐associated genes (e.g., Cxcl9, Ccl5, Ccl8) in mRNA‐treated groups (Figure S7e, Supporting Information). KEGG analysis revealed the upregulation of several anti‐tumor immune pathways (Figure S7f, Supporting Information), indicating TAM remodeling. Regarding neutrophils, we observed abundant infiltration in control NPs‐treated tumors (Figure 5k‐m). Cells in the dominant subcluster (Neu_0, Neu_1, Neu_2, and Neu_5) with high expression of Ccl3 and Cstb defined as tumor‐specific neutrophils,^[^ ^19^ ^]^ which were enriched in the NPs group and exert pro‐tumor effects with a similar expression pattern of Cd274, Vegfa, and Lgals3 (Figure 5l,m). Additionally, two subclusters (Neu_3, Neu_4) exhibited high levels of IFN‐stimulated gene expression (Figure 5l), likely resulting from the interaction with IFN‐γ^+^ cells.^[^ ^20^ ^]^ These findings suggest that the decreased infiltration of tumor‐promoting neutrophils may account for tumor growth inhibition in mRNA‐treated groups. Overall, our bulk RNA‐seq combined with scRNA‐seq analyses revealed that mOVA@NPs reshape the immunosuppressive TME toward a more proinflammatory state.

### 2.5 mHBsAg@NPs have a Strong Anti‐Tumor Effect in HBV Vaccination Model

**Figure 6:**
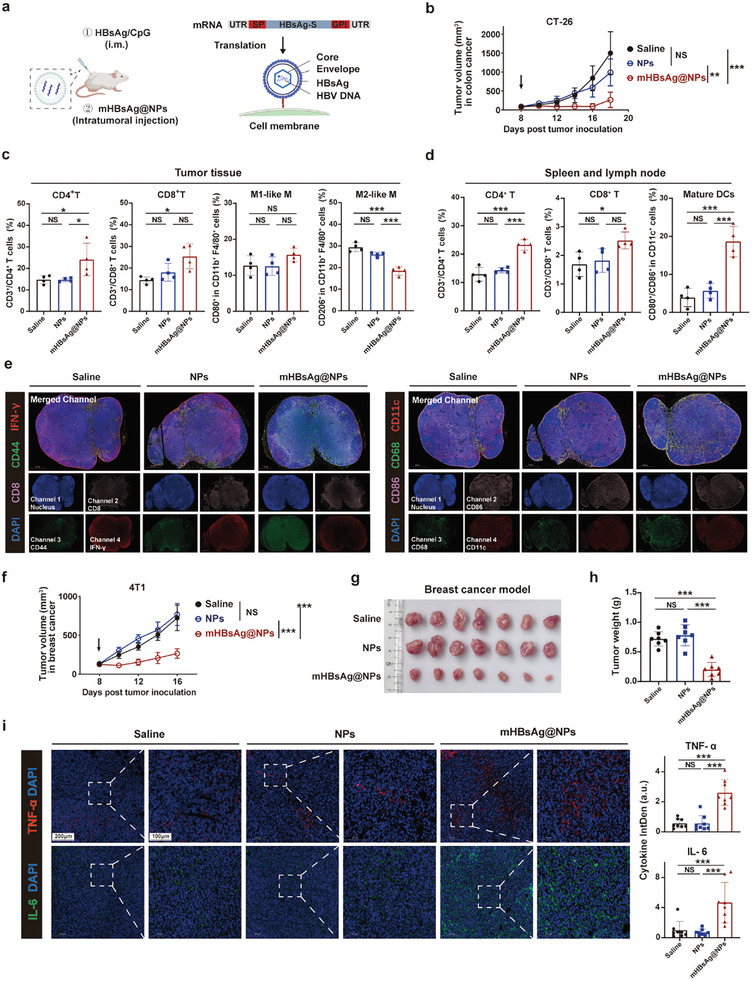
mHBsAg@NPs have a strong anti‐tumor effect in an HBV vaccination model. a) Schematic of intratumoral injection of mHBsAg@NPs. The mRNA consisted of a 5′ untranslated region (UTR), signal peptide (SP), coding sequence (CDS), C‐terminal GPI‐anchor, and 3′ UTR. The CDS could be translated to HBsAg. b) Average tumor volume curves for mouse models of colon tumors (*n*  =  7). c) Flow cytometric analysis of the levels of CD3^+^/CD8^+^ and CD3^+^/CD4^+^ T cells and the levels of M1‐TAM and M2‐TAM in colon tumor tissues (*n*  =  4). d) Flow cytometric analysis of the levels of CD3^+^/CD8^+^ and CD3^+^/CD4^+^ T cells in the spleen (*n*  =  4) and CD11c^+^/CD80^+^/CD86^+^ mature DCs in lymph nodes (LNs) (*n*  =  4). e) Immunofluorescence staining of inguinal LNs from different groups for analyzing the effector (IFN‐γ^+^, red) memory (CD44^+^, green) CD8^+^ (pink) T cells, and the activation (CD86^+^, pink) of macrophages (CD68^+^, green) and DCs (CD11c^+^, red). Scale bars represent 500 µm. f) Average tumor volume curves for mouse models of breast cancer tumors (*n*  =  7). g, h) Tumor images (g) and tumor weights (h). i) Immunostaining of tumors for TNF‐α (red)/IL6 (green) on day 16 after the indicated treatments in the 4T1 model. The signal intensity was quantified. Scale bars, 200 µm (low magnification) and 100 µm (high magnification). All data are presented as the mean ± SD. One‐way ANOVA with Tukey's multiple comparisons test was used for all statistical analyses. *P < 0.05; **P < 0.01; ***P < 0.001; NS, not significant.

Having confirmed the anti‐tumor effect of this mRNA vaccine strategy using OVA, we attempted to extend this strategy to clinical antigens. Hepatitis B virus (HBV) infection remains a long‐standing worldwide public health issue.^[^ ^21^ ^]^ Hepatitis B surface antigen (HBsAg) has been used as the commercial antigen for HBV vaccine for more than 30 years.^[^ ^22^ ^]^ We developed the mRNA vaccine using HBsAg as the antigen, referred to as mHBsAg@NPs. As shown in **Figure** **6**a, mice were initially vaccinated with HBsAg/CpG to establish the immune memory model, followed by intratumoral injection of mHBsAg@NPs to evaluate the anti‐tumor effect across different subcutaneous solid tumors.

In the colon cancer model, mice treated with mHBsAg@NPs exhibited significant inhibition of tumor growth (73.2%), with 2 of 7 mice achieving complete tumor regression (Figure 6b; Figure S8a,b, Supporting Information). Furthermore, we observed marked reductions in lung cancer metastasis and shrinkage of tumor cell nucleus (Figure S8c, Supporting Information). Similar to the effects observed in the OVA model, mHBsAg@NPs treatment resulted in an increase in tumor‐infiltrating CD3^+^/CD4^+^ and CD3^+^/CD8^+^ T cells, along with alteration in macrophage polarization (Figure 6c).

Considering that mRNA reprogramming leads to the presentation of epitopes on the surface of only a fraction of tumor cells, we hypothesized that strong anti‐tumor response resulted from the dissemination of endogenously presented antigens. To verify this, we examined both spleens and tumor‐draining lymph nodes (TdLNs) from each group via flow cytometric analysis. Following the administration of mHBsAg@NPs, the numbers of both T helper cells and effector cells within the spleen increased (Figure 6d). Additionally, DCs in TdLNs of mHBsAg@NPs‐treated mice were effectively activated, exhibiting 3.28‐fold higher levels of the maturation marker CD80 and CD86 compared to the control group (Figure 6d). Additionally, the TdLNs were collected for analyzing effector (IFN‐γ) elicitation of memory CD8 T cells and the activation (CD86) of DCs (CD11c) and macrophages (CD68) by immunofluorescence staining (Figure 6e). The results indicated that the mRNA vaccine induced a higher proportion of memory CD8 T cells, as well as activated DCs and macrophages in the draining LNs (Figure S9a,b, Supporting Information). Collectively, these findings imply that tumor clearance is reliant on the abundant endogenously presented antigens, that are further captured and processed by antigen‐presenting cells (APCs), thereby fostering the generation of a more diverse pool of T cells at the systemic level.

Given the strong association between HBV infection and hepatocellular carcinoma (HCC),^[^ ^23^ ^]^ we also evaluated the anti‐tumor effects in mouse models of HCC. As illustrated in Figure S10a‐d (Supporting Information), tumor proliferation was inhibited by 74.8% in the mRNA vaccine‐treated group, accompanied by a higher infiltration of T cells, particularly CD8 T cells (Figure S10e, Supporting Information). Additionally, we observed a notable elevation of the secretion of key inflammatory cytokines and effector molecules within the tumor, including TNF‐α, Granzyme B, and IFN‐γ, suggesting the activation of anti‐tumor responses (Figure S10f, Supporting Information). Similar therapeutic effects were observed in both the 4T1 (65.5%) and B16‐F10 (85.2%) cancer models (Figure 6f‐h; Figure S11a‐d, Supporting Information). Additionally, consistent TME markers for mHBsAg@NPs were identified, analogous to those observed in the OVA model (Figure S11e,f, Supporting Information). Quantification of cytokines further confirmed that the immunosuppressive TME was reshaped through the increase of cytokine secretion (Figure 6i; Figure S12a,b, Supporting Information). Finally, no obvious toxicity was observed in the vaccine‐treated group (Figure S12c‐e, Supporting Information). Taken together, all above results demonstrated that mHBsAg@NPs exert robust anti‐tumor effects in HBV‐vaccinated mice across various subcutaneous cancer models, from the local to the systemic level.

### 2.6 mSRBD@NPs Inhibit Tumor Growth in COVID‐19 Immune Model in a Dose‐Dependent Manner

**Figure 7:**
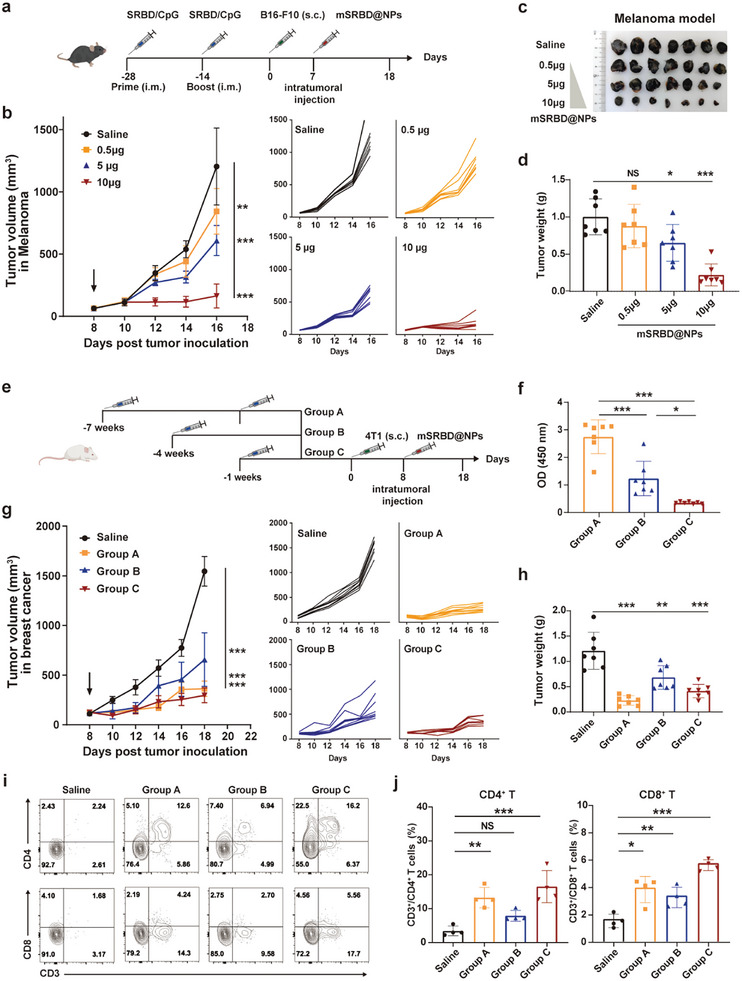
mSRBD@NPs inhibit tumor growth in COVID‐19 immune model in a dose‐dependent manner. a) Experimental timeframe for SRBD vaccination and intratumoral injection with different dosages of mSRBD@NPs in B16‐F10 tumor‐bearing mice. b) Average and individual tumor volume curves for a mouse model of melanoma (*n*  =  7). c, d) Tumor images (c) and tumor weights (d). e) Experimental timeframe for SRBD vaccination to obtain different levels of SRBD‐specific lgG in 4T1 tumor‐bearing mice. f) SRBD‐specific IgG antibody titer determined by ELISA in 4T1 cancer model (*n*  =  6). g) Average and individual tumor volume curves for a mouse model of breast cancer (*n*  =  7). h) Tumor weight of a mouse model of breast cancer (*n*  =  7). i, j) Flow cytometric analysis of the levels of CD3^+^/CD4^+^ and CD3^+^/CD8^+^ T cells in tumor tissue from a mouse model of breast cancer with different levels of immunity (*n*  =  4). All data are presented as the mean ± SD. One‐way ANOVA with Tukey's multiple comparisons test was used for all statistical analyses. *P < 0.05; **P < 0.01; ***P < 0.001; NS, not significant.

Given the importance of the global COVID‐19 pandemic, we further extended this mRNA vaccine strategy to the antigens of SARS‐CoV‐2.^[^ ^24^ ^]^ We developed the mRNA vaccine using the receptor‐binding domain (RBD) of SARS‐CoV‐2, referred to as mSRBD@NPs. To verify whether anti‐tumor effects were dose‐dependent, we set different concentration gradients for comparative analysis (**Figure** **7**a). mSRBD@NPs provided dose‐dependent anti‐tumor protection (Figure 7b‐d). Mice treated with 10 µg mSRBD@NPs showed significant tumor growth inhibition (88.9%), while the anti‐tumor effects were less strong in mice treated with a dose of 5 µg. The lower dosage of 0.5 µg was unlikely to suppress tumor growth (Figure 7b‐d). These observations suggest that the antigen expression level during in situ reprogramming is crucial for tumor suppression. We also provided consistent TME markers for mSRBD@NPs (Figure S13a‐b, Supporting Information). Weaker tumor growth inhibition was observed in a colonic cancer model (70.5%) (Figure S14a‐e, Supporting Information). mSRBD@NPs promoted recruitment of tumor‐infiltrating T cells, macrophage polarization, and DC maturation, (Figure S14f‐g, Supporting Information). However, antigen‐specific cytotoxic T cells and helper T cells among splenocytes treated with mSRBD@NPs showed small but insignificant differences with control (Figure S14h, Supporting Information). These results indicate that mSRBD@NPs showed limited activation of the systemic anti‐tumor response in our SARS‐CoV‐2 vaccination model, probably due to the weaker immunogenicity of the antigens.

To further evaluate the correlation between the level of memory immunity and the anti‐tumor effect, we generated different levels of antigen‐specific antibodies in mice through different vaccination timeframes (Figure 7e). Group A harbored the highest levels of SRBD‐specific antibodies, followed by group B, and group C had the lowest levels (Figure 7f). As expected, the strongest tumor inhibition was observed in group A, which could have been attributable to the second boost (Figure 7g,h). The infiltration of T cells into tumors was consistent with the effect of tumor inhibition (Figure 7i,j). Lastly, mSRBD@NPs exhibited no obvious toxicity (Figure S14i,j, Supporting Information).

## 3 Discussion

The present study has developed a universal strategy of anti‐tumor mRNA vaccine by harnessing “off‐the‐shelf” immunity. We used the model antigen ovalbumin and two clinical antigens (HBsAg and SARS‐CoV‐2 SRBD) to prepare mRNA vaccines (mX@NPs) that could markedly inhibited tumor growth across three solid tumor models. Our findings provide not only proof of concept for the universal anti‐tumor mRNA therapy, but also mechanistic insights in spontaneous cancer regression.

### 3.1 A Universal Anti‐Tumor mRNA Vaccine Strategy

Traditional universal cancer vaccines typically utilize tumor‐associated or tumor‐specific antigens, which are highly expressed in tumor tissues.^[^ ^4^ ^]^ By contrast, our study presents a novel design that employs mRNA encoding tumor‐unrelated antigens. These antigens are not expressed by intrinsic tumor tissue but are designed to serve as the “labels” for in situ reprogramming. Therefore, using mRNA vaccine technology, we achieve reactivating pre‐existing immune memory to specific antigens. This mRNA‐based strategy provides a novel therapeutic approach for cancer immunotherapy.

Our mRNA‐based strategy has several critical advantages. First, it requires no customized screening, neoantigen identification, or lengthy production pipeline, thus enables large‐scale production and rapid deployment. And for clinical use, this approach has potential to shorten treatment timelines and be beneficial for a wider range of patients.^[^ ^7^, ^25^ ^]^ Moreover, this mRNA‐based strategy provides a broadly applicable intervention framework suitable for various scenarios. First, the choice of universal antigen is flexible, which is advantageous for clinical applicability. Preferentially selected clinical antigens are derived from pathogens that are highly prevalent in humans. Theoretically, using antigens from HBV and SARS‐CoV‐2 would be applicable to the majority of patient populations worldwide. In particular, universal HBV immunization program has been used in China for over two decades.^[^ ^26^ ^]^ Additionally, the use of multiple‐antigen combinations would likely increase the efficacy of attack by T cells.^[^ ^27^ ^]^ As the majority of the T cell epitope repertoire against variants are highly preserved (>80%) in natural conditions of infection,^[^ ^17a^ ^]^ We hypothesized that this universal strategy may produce more or less boosted anti‐tumor response in different infected populations, regardless of the variant with which they were infected. Third, generation of immune memory T cells is not limited to the scenario described in this study, it may be extended to either natural pathogen infections or immunity generated by viral mimics. Additionally, although we only evaluated the therapeutic efficacy of this mRNA‐based vaccine strategy in three subcutaneous cancer models, we speculate that it may exert similar effects on other types of solid tumors. Overall, our study provides conceptual validation for this universal anti‐tumor mRNA‐based strategy.

Although our findings demonstrated the potency of tumor inhibition, there were still some disadvantages and limitations. One clinical obstacle is the limited feasibility of intratumoral injection, which requires the optimization of delivery systems. Additionally, continuous antigen exposure during our mRNA vaccine strategy may lead to T cell functional exhaustion and immune evasion. This challenge may be overcome by combination with other immunotherapies, such as immune checkpoint inhibitors.

### 3.2 A Mechanistic Model Underlying this mRNA Vaccine Strategy

**Figure 8:**
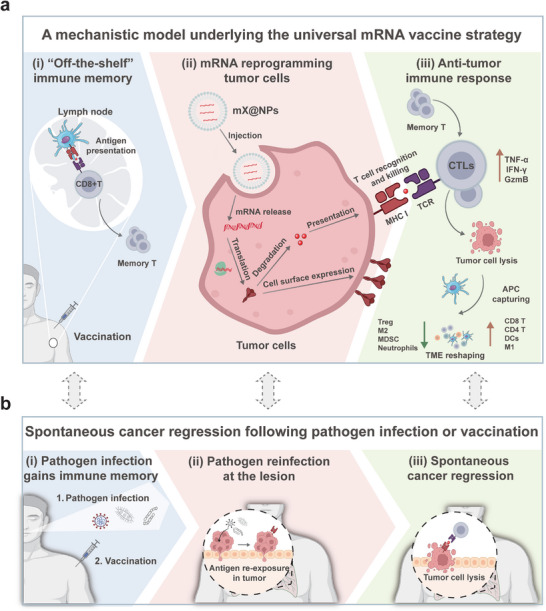
Schematics of proposed model for this anti‐tumor mRNA vaccine strategy. a) Schematic of the application of mX@NPs for reprogramming tumor cells and priming “off‐the‐shelf” immunity. (i) In response to vaccination, antigen‐specific CTLs were first generated in lymph nodes and then presented as persistent memory T cells. (ii) mRNA vaccine (mX@NPs) acts to reprogram tumor cells through presentation of universal antigens on the surface of tumor cells. (iii) Preexisting memory T cells are expanded and migrate to the lesion where they exert recognition and killing function, causing tumor cell apoptosis with abundant antigen release, subsequently leading to systemic immune activation and TME reshaping. b) The present study largely recapitulated the clinical scenario of spontaneous regression of cancer following pathogen infection or vaccination. (i) Pathogen (viruses, bacteria, fungi, etc.) infection or vaccination resulted in preexisting memory immunity within cancer patients. (ii) Upon intralesional reexposure to the same antigen from the above pathogen, antigen‐specific memory T cells are recruited to the neoplastic lesion to lyse tumor cells. (iii) The anticancer responses subsequently lead to spontaneous cancer regression. The bidirectional arrows represent the correspondence between our mRNA vaccine strategy and spontaneous cancer regression.

Given our in vivo evidence with bulk and scRNA‐seq analyses, we propose a model underlying our strategy, which is dominated by the activation of antigen‐specific CTLs (**Figure** **8**a). In response to vaccination, antigen‐specific CTLs are first generated in lymph nodes and then persisted as long‐lived memory T cells with high vigilance.^[^ ^13^, ^28^ ^]^ We hypothesize that the mRNA vaccines (mX@NPs) developed in the present study function to reprogram tumors by presenting antigens on the cell surface via an endogenous MHC‐I pathway (Figure 8a, left panel), thereby rendering marked tumor cells recognizable by activated memory T cells. They then exert their killing function by secreting effector and cytolytic molecules, such as IFN‐γ, TNF‐α, and granzyme B (Figure 8a, middle panel). After tumor apoptosis, endogenously presented tumor antigens are further captured and processed by tumor‐infiltrating APCs. This process fosters the generation of a more diverse pool of effector T cells at the systemic level (Figure 8a, right panel). Thereby, this strategy subsequently reshapes the TME toward a more proinflammatory state. Our mRNA vaccine strategy demonstrates stronger tumor growth inhibition in melanoma and suboptimal effects in breast cancer. The differences may be attributable to the microenvironment and immune characteristics of the different tumor types, including the capacity for endogenous MHC‐I presentation and T cell infiltration.^[^ ^29^ ^]^ Similar to our strategy, delivery of viral epitope peptides to the tumor cell surface has been attempted. The majority of such studies have utilized peptides without cellular internalization.^[^ ^14^, ^15^ ^]^ However, due to the size restriction of MHC‐I, these viral epitope peptides are designed with lengths of only 8–11 amino acid residues, resulting in a limited CTL response.^[^ ^30^ ^]^ By contrast, the mRNAs encoding full‐length antigens used in the present study allow the simultaneous presentation of multiple epitopes.

### 3.3 Mechanistic Insights into Spontaneous Cancer Regression

Reports of spontaneous cancer regression can be traced back over a hundred years. Anecdotal reports have documented confirmed cancer patients who contracted COVID‐19 or another virus and experienced SR of certain tumors.^[^ ^9^ ^]^ Previous studies on this have revolved around three main conjectures. First is the priming hypothesis, which proposes that SARS‐CoV‐2 infection induces a severe acute inflammatory response through innate hyperactivation of immunity.^[^ ^9^ ^]^ The second hypothesis involves the cross‐priming of pathogen‐specific T cells with tumor antigens.^[^ ^10^, ^31^ ^]^ The third hypothesis proposes that certain viruses act as oncolytic virus targeting host cells and mediating cell death through lytic mechanisms.^[^ ^8^, ^11^, ^32^ ^]^ However, due to the lack of clinical cases and systematic experimental validation, the intricate mechanisms underlying cancer regression have yet to be fully elucidated.

Our findings provide mechanistic insights in echoing the long‐standing puzzle of spontaneous cancer regression (Figure. 8b). Coincidentally, the present study largely recapitulated the scenario of spontaneous regression of cancer following pathogen infection or vaccination. Specifically, the process by which patients harbor memory immunity through pathogen infection or vaccination corresponds to the process of building immune memory in mouse models (Figure 8a,b, left panel). Next, the pathogen reinfection event at the lesion may correspond to the reprograming of tumors to reactivate pre‐existing antigen‐specific memory T cells in our mRNA vaccine strategy (Figure 8a,b, middle panel). Finally, the resulting spontaneous cancer regression may correspond to the anti‐cancer immune response elicited by mRNA vaccination (Figure 8a,b, right panel). In this scenario, we speculate that spontaneous cancer regression may result from the activation of pre‐existing memory immunity. This hypothesis is further supported by observations that pathogen‐specific CD8^+^ T cells naturally infiltrate various tumors,^[^ ^15^, ^33^ ^]^ suggesting that pathogen reinfection occurs at the lesions. Moreover, tumors exhibited heightened susceptibility to viral infection, possibly due to the preference for virus replication.^[^ ^34^ ^]^ In summary, this study substantially advances our understanding of the mechanisms underlying spontaneous cancer regression following pathogen infection or vaccination.

### 3.4 Limitations for this Study

To circumvent the limitation about targeting and specificity, further research focusing on tumor‐specific delivery systems and antigen selection is required. The expression of universal antigens sacrifices specificity to some extent. Additionally, loss of tumor cell MHC I presenting could possibly lead to antigen exhaustion and immune escape. The combination with other immunotherapy methods (such as immune checkpoint inhibitors) seems to provide effective solutions. For tumor tissue‐specific antigen expression, microRNA‐mediated Trojan horse strategies could be utilized to alleviate off‐target expression in local therapy.^[^ ^35^ ^]^

## 4 Experimental Section

### Cell Lines and Animals

B16‐F10, 4T1, and CT‐26 cells were purchased from Hai Xing Biosciences (Suzhou, China). Hepa1‐6 cell was a gift from J. Wu (Zhejiang University). B16‐F10, CT‐26, and Hepa1‐6 were cultured in DMEM medium containing 10% fetal bovine serum (FBS) and 1% penicillin/streptomycin. 4T1 was cultured in RPMI 1640 medium containing 10% fetal bovine serum and 1% penicillin/streptomycin.

C57BL/6, Balb/c, and OT‐1 female mice (4‐6 weeks old) were obtained from Qi Zhen laboratory animal Tech Co. Ltd., China and used for anti‐tumor effect evaluation. The animals were randomly assigned into cages in a room maintained at 22 ± 2.0°C and 50 ± 10% humidity and subjected to a 12 h light/12 h dark cycle before the study. All mice were given access to a standard diet and tap water. All animal experiments were performed in accordance with the China Public Health Service Guide for the Care and Use of Laboratory Animals. All animal experiment protocols were approved by the Animal Ethical Committee of Zhejiang University.

### Synthesis of mRNA

For OVA mRNA, it encodes for the immunoglobulin kappa chain signal peptide (IgK), OVA antigen from Gallus gallus (MF321659.1), and C terminus of the OVA construct was linked to the human decay‐accelerating factor (DAF) responsible for the attachment of a glycosylphosphatidylinositol (GPI) anchor.^[^ ^16^, ^36^ ^]^ The target fragment was connected to vector pUC‐57 by homologous recombination. For HBsAg mRNA and SRBD mRNA, the antigens were respectively replaced by spike protein from Hepatitis B virus strain s2 reverse transcriptase gene (MK213856.1) and receptor binding domain of surface glycoprotein from severe acute respiratory syndrome coronavirus 2 (YP_009724390) (Table S3, Supporting Information). The whole target fragments were then connected to linearized template vectors (Takara Biotech Co. Ltd., China) by seamlessly cloning to construct plasmids for downstream in vitro transcription reactions.

mRNA was synthesized using standard in vitro transcription methods. Plasmids encoding specific antigens were digested overnight with *HindIII* to obtain a linearized template. mRNA was then produced using the T7 High Yield RNA Transcription Kit with N¹‐Me‐Pseudo UTP completely substitution (Vazyme Biotech Co. Ltd., China) following manufacturer instructions.^[^ ^37^ ^]^ Then mRNA was purified using EasyPure RNA Purification Kit (Transgen Biotech Co. Ltd., China) and capped using Vaccinia Capping Enzyme and 2’‐O‐methyltransferase (Vazyme Biotech Co. Ltd., China) to obtain a Cap‐1 structure. mRNA was further purified with the column. All mRNAs were analyzed by agarose gel electrophoresis and stored frozen at −80 °C.

### Preparation and Characterization of mOVA@NPs

The mOVA@NPs formulation was based on the disclosed prescription by Moderna.^[^ ^38^ ^]^ Specifically, ionizable lipid SM102, cholesterol CHO‐hP, DSPC, and DMG PEG‐2000 (AVT Co. Ltd., Shanghai, China) were mixed at a mass ratio of 50:10:38.5:1.5 with absolute ethanol. Three volumes of mRNAs [1:25 (w/w) mRNA to lipid, nitrogen/phosphate (N/P) ratio = 6] were suspended in sodium citrate buffer (pH 4.5). The two solutions were then mixed by rapid pipetting and transferred into dialysis overnight at 4 °C against PBS. The particles in PBS were used for further characterization. The morphology of mX@NPs was imaged using JEM‐1400 flash transmission electron microscopy (TEM). The size distribution and zeta potential of mX@NPs were determined by dynamic light scattering (DLS) (Zetasizer Nano ZS90, Malvern Instruments, Malvern, UK).

### mOVA@NPs Loading and Stability Study

To test the loading ability, naked mRNA, control NPs, and mOVA@NPs were incubated at room temperature for 10 min, A 1.5% agarose gel electrophoresis analysis was performed for 20 min at 80V to assess the loading capacity by band position under ultraviolet light. To evaluate the stability of mOVA@NPs, Cy3‐mOVA@NPs were incubated with RNase A for various durations (0, 0.5, 1, 3, 6, 12, 24 h). After that, fluorescence intensity was detected using a multifunction enzyme marker (FlexStation 3, Molecular Devices, US).

### Cellular Uptake and Lysosomal Escape of mOVA@NPs

To monitor the cellular uptake of mOVA@NPs, 4 × 10^5^ cells were seeded in confocal plates per well and incubated overnight at 37°C, the old cell medium was then removed, and fresh medium containing Cy3‐mOVA@NPs (100 µg mL^−1^) was added. Cells were cultured for 0, 0.5, 4, 6,18 h, respectively. The cells were then washed with DPBS for three times and fixed with 4% paraformaldehyde. For detection, the cells were stained with Lyso‐Tracker Green (Solarbio Life Sciences, China) and DAPI, and then imaged by CLSM (Leica FLUOVIEW FV3000 CLSM, Olympus, Japan) to analyze the cellular uptake and lysosomal escape of mOVA@NPs.

### Membrane localization of mRNA

Cells were inoculated at a density of 1 × 10^5^ cells per well in confocal culture plates and cultured in medium containing 10% fetal bovine serum. When the cells reached 60%–70% fusion, mOVA@NPs (with mcherry label) were added to each well and incubated for 24 h. The cells were washed with DPBS and fixed with 4% paraformaldehyde at room temperature for 15 min. After the DPBS wash, 3,3’‐dioctadecyloxacarbocyanine perchlorate (DIO) was added and cells were incubated at 37 °C for 20 min, protected from light. Finally, the cells were stained with DAPI and washed with DPBS, finally imaged using CLSM (Leica FLUOVIEW FV3000 CLSM, Olympus, Japan).

### In Vitro Cytotoxicity Evaluation

B16‐F10, CT‐26, and 4T1 cells were inoculated in 96‐well plates at a density of 1 × 10^4^ per well, and cultured in medium containing 10% fetal bovine serum for 24 h. The cells were then incubated for 24 h using control NPs and mOVA@NPs at concentrations of 0, 1, 10, 25, 50, 100, 250, 500, and 1000 ng mL ^−1^. Empty material groups were set up at corresponding concentrations in the absence of mRNA, and equal amounts of DPBS were added as blank control groups. Finally, 10 µL of Cell‐Counting‐Kit‐8 (APExBIO Tech, China) solution was added to each well, and cell viability was detected at 450 nm after incubation for 1 h.

### In‐Vitro mRNA Expression

B16‐F10 cells were first seeded in a six‐well plate at a density of 4 × 10^5^ cells per well overnight one day before transfection. The cells were then treated with 1 mL of medium containing saline, NPs, and mOVA@NPs (with mcherry tag) for 48 h. After that, cells were washed with DPBS for three times and fixed with 4% paraformaldehyde for 20 min at room temperature. Finally, cells were stained with DAPI for 10 min and imaged using CLSM. Alternatively, cells were collected in a 1.5 mL tube, resuspended in 200 µL of FACS buffer. The analysis was using flow cytometry (MoFlo XDP, Beckmancoulter, US). In‐vitro OVA expression was quantified by ELISA in both the supernatants and lysates of cells.

### In‐Vivo Expression and Bioluminescence Imaging

CT‐26 tumor cells were implanted subcutaneously into Balb/c female mice (4‐6 weeks old) to establish the colon cancer model. When the tumor volume reached 50–100 mm^3^, the mice were intratumorally injected with NPs encapsulating 10 µg of luciferase‐encoding mRNA (mLuciferase@NPs) and NPs for control. After 6, 12, and 24 h, 200 µL of luciferin at a concentration of 15 mg mL^−1^ was intratumorally injected into the mice. After 10 min, the bioluminescence from the mice was imaged using the In Vivo Imaging System (IVIS Spectrum, Caliper, US).

### In‐Vitro CD8^+^ T Cell Expansion, Activation, and Killing

The spleen of OT‐1 mice was sterilized in the biosafety cabinet and centrifuged at 1000 rpm after passing 45 µm cell strainers. After lysis with red blood cell lysate for 3 min, the splenocytes were resuspended in DPBS. CD8a^+^ T Cell Isolation Kit (Beaver Biosciences Inc.) was used to obtain a purified CD8^+^ T cell suspension. After that, 1 × 10^6^ purified naive T cells were seeded in a 24‐well plate, and 25 µL Dynabeads Mouse T‐activator CD3/CD28 magnetic beads (ThermoFisher Scientific Inc.) and 30 U mL^−1^ IL‐2 were added. The T cells were incubated at 37 °C for 72 h. B16‐F10 cells were seeded into a 24‐well plate at a density of 5 × 10^4^ cells per well overnight. The cells were then treated with 1 mL of medium containing DPBS for control and mOVA@NPs for transfection 24 h. Activated CD8^+^ T cells were added at a specific effect‐to‐target ratio and incubated for 12 h. After incubation, tumor cells were collected and analyzed using ANNEXIN V‐FITC/PI kit (Solarbio Science Technology Co., Ltd.), and CD8^+^ T cells were collected to evaluate IFN‐γ^+^ and Granzyme B expression using flow cytometry.

### In‐Vitro Stimulation of DCs

BMDCs were isolated from the bone marrow of female C57BL/6 mice. Briefly, single‐cell suspensions were prepared, passed through 45 µm cell strainers, and cultured in RPMI 1640 complete medium supplemented with 20 ng mL^−1^ GM‐CSF and 10 ng mL^−1^ IL‐4 (Peprotech) for one week. B16‐F10 cells were pretreated with PBS (Control), NPs, or mOVA@LNPs for 12 h, and then co‐cultured with activated CD8^+^ T cells from OT‐1 mice for an additional 12 h. Following this, the co‐culture was extended for 48 h with BMDCs. The maturation biomarkers for DCs (CD80 and CD86) were assessed by flow cytometry, gated on CD11c^+^ cells.

### Tumor Treatment

The effects of three mRNA‐based cancer vaccines (mOVA@NPs, mHBsAg@NPs, mSRBD@NPs) in three different cancer models (melanoma, colon cancer, breast cancer) were evaluated. Specifically, for advanced immune model, immunize individual mice with protein vaccine (Antigen/CpG/Adjuvant) dissolved in 100 µL saline. The prime vaccination was four weeks ahead of schedule and the boost was two weeks in advance. At day 0, to establish the subcutaneous melanoma and breast cancer model, 1 × 10^6^ tumor cells in 200 µL DPBS were subcutaneously injected into the right flank of C57BL/6 or Balb/c mice. When the tumor volume reached 50–100 mm^3^, 100 µL of saline containing 10 µg of mX@NPs were intratumorally injected, along with saline and NPs as controls. For instant immune model, 1 × 10^6^ tumor cells were first subcutaneously injected to mice. After 4 days, individual mice were immunized with a vaccine (Antigen/CpG/Adjuvant) dissolved in 100 µL saline. When the tumor volume reached 50–100 mm^3^, 10 µg of mX@NPs were administered. To evaluate the anti‐tumor effect of mRNA vaccine, the tumor volumes of mice were recorded every two days according to the following formula: ½ (length × width × width). The weight of mice was also monitored every two days. The mice were euthanized around day 20, and the tumor tissue, spleen, and tumor‐draining lymph nodes were isolated to further examine the percentage and phenotype of immune cells.

### Flow Cytometry for Immune Cells and Antibodies

Antibodies for flow cytometry analysis were purchased from BioLegend Inc. The tumor tissues and lymph nodes were cut into pieces and added to RPMI 1640 media containing 10% FBS, collagenase IV (1 mg mL^−1^), hyaluronidase (0.2 mg mL^−1^), and DNase (0.1 mg mL^−1^) for 1 h of digestion in a 37 °C water bath. The tissue suspensions were then passed through a 45 µm filter membrane to create single‐cell suspensions. After erythrolysis, cells were blocked with TruStain FcX™ (anti‐mouse CD16/32) antibody for 20 min, and then stained on ice with fluorescence‐conjugated antibodies for 30 min. After staining, cells were resuspended with 200 µL of FACS buffer and ultimately evaluated by flow cytometry (CytoFLEX, Beckmancoulter, US) and analyzed using FlowJo software. Antibodies used in this study are listed in Table S2 (Supporting Information). The gating strategies for T cells, macrophage, natural killer cells, B cells, myeloid‐derived suppressor cells, and dendritic cells in this study are shown in Figures S15 and S16 (Supporting Information).

### Splenocyte Restimulation with Antigenic Peptides

A total of 1 × 10^7^ splenocytes per mouse were seeded into 24‐well plates after pulverization and then passed through 0.45 µm filter membranes. The cell medium consisted of RPMI1640 with 10% FBS, 1% penicillin/streptomycin, non‐essential amino acids (1x), HEPES (20mM final), and β‐mercaptoethanol (50 µM final). Splenocytes were stimulated with 20 ng of antigenic peptides per well. The plates were then incubated at 37 °C for 43 h. Brefeldin A was added for another 5 h incubation, and the splenocytes were collected for flow cytometry.

### ELISA for Antibody Titer

The antigen‐specific antibody titer was measured using indirect ELISA kit from Solarbio Life Sciences (China). High‐binding ELISA plates were coated with 100 µL of OVA at 500 ng mL^−1^ in a coating buffer at 4 °C overnight. The same procedure was applied for HBsAg and SARS‐CoV‐2 SRBD. The plates were then washed with PBS containing 0.5% Tween‐20 for three times and blocked by 5% BSA solution for 2 h. The diluted serum (1:10 000 dilution) was added to the plates and incubated for 2 h at 37°C. After washing four times, they were incubated with 1:5000‐diluted HRP anti‐IgG (H+L) antibodies, HRP anti‐IgG1 antibodies, and HRP anti‐IgG2a antibodies for 1 h. The plates were washed four additional times and incubated with 100 µL of 3,3’,5,5’‐tetramethylbenzidine substrate (TMB). The reaction was stopped with 0.16 M sulfuric acid solution. The optical density at 450 nm was measured using a Multimode Plate Reader (VICTOR Nivo, Thermo Fisher, US).

### Immunofluorescence and H&E Staining for Tissues

After tumor treatment, the mice were euthanized. The tumor tissues, lymph nodes, and various organs (lung, heart, liver, kidney, and spleen) were collected and fixed in 4% paraformaldehyde overnight. All organs were embedded in paraffin and sliced to a thickness of 5 µm. Then sections were then stained with H&E to detect potential pathological changes. For immunofluorescence staining, samples were incubated with different fluorescently labeled antibodies (CD3, CD8, CD4, FOXP3, TNF‐α, and IL‐6 in tumors; CD8, CD44, IFN‐γ, CD68, CD86, and CD11c in lymph nodes) and slides were imaged using a digital slicing scanner (3D Histech).

### Determination of Serum AST and ALT Levels

Blood from Saline, NPs, and mX@NPs‐treated mice was collected from the orbital sinus. Serum was obtained by centrifuging at 3000 rpm for 20 min. Serum aspartate transaminase and alanine transaminase levels were determined using a colorimetric assay.

### Cytokine Assay

Briefly, tumor tissue was homogenized, and the cell supernatants from each treatment groups were collected for cytokine assays using ELISA kit from Solarbio Life Sciences (China). Serum was obtained by centrifuging at 3000 rpm for 20 min for cytokine assays. The selected cytokines included TNF‐α, IFN‐γ, Granzyme B, IL‐6, IL‐4, and IL‐2.

### Quantitative Real‐Time PCR

Total RNA was extracted from cells using Trizol (Invitrogen) according to the manufacturer's protocol. RNA was reversely transcribed using the HiScript II 1st Strand cDNA Synthesis Kit (Vazyme Biotech Co. Ltd., China). The diluted complementary DNA (cDNA) was then used with HiScript III RT SuperMix for qPCR (Vazyme Biotech Co. Ltd., China) and gene‐specific primers. The reactions were performed in the Bioer 9600 FQD‐96A System. Beta‐actin was used as a housekeeping control. Primers used in this study are listed in Table S1 (Supporting Information).

### RNA Extraction Library Construction and Sequencing

Total RNA was extracted using Tirol (Thermo Fisher, 15596018) following the manufacturer's procedure. After total RNA was extracted, mRNA was purified from total RNA using Dynabeads Oligo (dT) (Thermo Fisher, CA, USA) with two rounds of purification. Following purification, the mRNA was fragmented into short fragments using divalent cations under elevated temperature (NEB, cat. e6150, USA). Cleaved RNA fragments were then reverse‐transcribed to create cDNA using SuperScript™ II Reverse Transcriptase (Invitrogen, cat. 1896649, USA), which was subsequently used to synthesize U‐labeled second‐stranded DNAs with E. coli DNA polymerase I (NEB, cat.m0209, USA), RNase H (NEB, cat.m0297, USA), and dUTP Solution (Thermo Fisher, cat. R0133, USA). An A‐base was added for ligation to the indexed adapters. Each adapter contained a T‐base overhang for ligating the adapter to the A‐tailed fragmented DNA. Dual‐index adapters were ligated to the fragments, and size selection was performed with AMPureXP beads. After treatment with the heat‐labile UDG enzyme (NEB, cat.m0280, USA), the ligated products were amplified. The average insert size for the final cDNA libraries was 300±50 bp. Finally, we performed 2 × 150 bp paired‐end sequencing (PE150) on an Illumina Novaseq™ 6000 (LC‐Bio Technology CO., Ltd., Hangzhou, China). The cDNA libraries constructed were then sequenced run with Illumina Novaseq^TM^ 6000 sequence platform. Using the Illumina paired‐end RNA‐seq approach, the transcriptome was sequenced, generating a total of millions of 2 × 150 bp paired‐end reads. Reads were further filtered using Cutadapt (https://cutadapt.readthedocs.io/en/stable/). The sequence quality was verified using FastQC (http://www.bioinformatics.babraham.ac.uk/projects/fastqc/, 0.11.9) including the Q20, Q30, and GC‐content of the clean data.

### Single‐Cell Dissociation

Mice were first vaccinated with OVA protein according to the “prime and boost” timeframe. Then mice were injected subcutaneously with B16‐F10 cells in 200 µL of PBS. When the tumor volume reached 50–100 mm^3^, mice were intratumorally administered with NPs and mOVA@NPs (3 mice each group). Tumor tissues were harvested three days later. Then a scRNA‐seq was performed at the laboratory of Lc‐Bio Technologies (Hangzhou) Co., Ltd. The obtained tumor tissues were minced into 0.5 mm^2^ pieces and digested in a dissociation solvent (0.35% collagenase IV, 2 mg mL^−1^ papain, 120 U mL^−1^ DNase I) for 20 min. Following digestion, the specimens were filtered through 70‐micron cell sieves to achieve single cell suspensions. The cell suspensions were then treated with erythrocyte lysis buffer (MACS 130‐094‐183,10X) for 10 min. After lysis, the cells were centrifuged, and the cell precipitate were resuspended in flow cytometry buffer. Add 100 µL of Dead Cell Removal MicroBeads (MACS130‐090‐101) for 15 min incubation. At the end of incubation, binding buffer was added to MS Columns (130‐042‐201) to remove reagents. APC Anti‐mouse CD45 (BioLegend, catalog no. 147708) was employed to label the cell surface. The cells were labeled for 45 min at 4 °C, protected from light. Subsequently, CD45^+^ cells were extracted and analyzed from each specimen. Cell viability was assessed using trypan blue staining method, with a required viability of > 85%. The number of cells was counted using a Countess II Automated Cell Counter, and the cell concentration was adjusted to 700–1200 cells µL^−1^.

### Single‐Cell Sequencing

Single‐cell suspension was added to the 10x Chromium chip according to the instructions for the 10X Genomics Chromium Single‐Cell 3’ kit (V3), with the expectation of capturing 8000 cells. Libraries were run on the HiSeq4000 for Illumina sequencing. cDNA amplification and library construction were performed according to standard protocols. Libraries were sequenced by LC‐Bio Technology (Hangzhou, China) on an Illumina NovaSeq 6000 sequencing system (double‐end sequencing, 150 bp) at a minimum depth of 20 000 reads per cell.

### Statistical Analysis of Single‐Cell RNA Data

Results from Illumina sequencing offline were converted to FASTQ format using bcl2fastq software (version 5.0.1). The scRNA‐seq data were compared to reference genome using CellRanger software, and cellular and individual cellular 3’ end transcripts were identified and counted in the sequenced samples. (https://support.10xgenomics.com/single‐cell‐gene expression/software/pipelines/latest/what‐is cell‐ranger, version 7.0.0). The output CellRanger expression profile matrix was loaded into Seurat (version 4.1.0) for filtering low‐quality cells from scRNA‐seq data, followed by downscaling and clustering. Filtering low cell quality thresholds: number of genes expressed per cell >500, mitochondrial genes expressed in <25% of cells. Cells were projected into 2D space using UMAP. These steps include: Calculating gene expression values using the LogNormalize method of Seurat's “NormalizeData” function; Performing principal component analysis (PCA) using the normalized expression values, using the top 20 PCs for clustering, and Findcluster analysis; Analyze the marker genes of each cluster based on Findallmarker, and the marker genes were selected based on the following criteria: expressed in more than 10% of cells in each cluster, P value ≤0.01, gene expression ploidy logFC ≥ 0.26. Hypergeometric testing was used to perform GO and KEGG enrichment analysis on the differential genes of each cluster obtained from Findallmarker analysis relative to other clusters.

### Statistical Analysis

All experiments were repeated at least three times. Statistical data were graphed and analyzed using GraphPad Prism version 8.0.2 (GraphPad Software, Inc., USA) and Image J. Quantitative results are presented as means ± SDs. Sample sizes for each statistical analyses were noted in the figure legends. For comparisons between two groups, a two‐tailed unpaired Student's t‐test was employed. Comparisons among multiple groups were performed using one‐way analysis of variance (ANOVA) with a Tukey's multiple comparisons test. *P < 0.05; **P < 0.01; ***P < 0.001; NS, not significant. Error bars in the graphical representations correspond to the means ± SDs.

### Data Availability

The raw sequence data of bulk RNA‐seq data generated in this study have been deposited in the Sequence Read Archive (SRA) database with accession numbers PRJNA1045070 and PRJNA1178826. The raw sequence data of single‐cell RNA‐seq data generated in this study have been deposited in the SRA database with accession numbers PRJNA1045076. All data needed to evaluate the conclusions in the paper are present in the paper and/or the Supplementary Materials.

## Conflict of Interest

The authors declare no conflict of interest.

## Supporting information



Supporting Information

## Data Availability

The data that support the findings of this study are available from the corresponding author upon reasonable request.
