# Supplementary material for: A Universal Strategy of Anti‐Tumor mRNA Vaccine by Harnessing “Off‐the‐Shelf” Immunity
Source: Adv Sci (Weinh). 2025 Jan 6;12(8):2401287. doi: 10.1002/advs.202401287 (PMC11848573; doi:10.1002/advs.202401287)
Supplement: Supplementary file 1 — Supporting Information [file ADVS-12-2401287-s001.pdf]

ADVANCED SCIENCE
Open Access

Supporting Information

for *Adv. Sci.*, DOI 10.1002/advs.202401287

A Universal Strategy of Anti-Tumor mRNA Vaccine by Harnessing "Off-the-Shelf" Immunity

*Jiayan Fu, Shuangqi Wu, Nengcheng Bao, Lili Wu, Huiru Qu, Zhechao Wang, Haiyang Dong, Jian Wu and Yongfeng Jin**

Supporting Information

A universal strategy of anti-tumor mRNA vaccine by harnessing "off-the-shelf" immunity

*Jiayan Fu[1,2], Shuangqi Wu[1,2], Nengcheng Bao[1,2], Lili Wu[1,2], Huiru Qu[1,2], Zhechao Wang[1,2], Haiyang Dong[1,2], Jian Wu[3], Yongfeng Jin[1,2]**

This PDF file includes:

Tables S1 to S3

Figure S1 to S16

Table S1. Specific primers used for RT-qPCR in this study.

Gene primers	5'-3' sequence
**H2-Ab1-F**	CTGTGGTGGTGCTGATGGT
**H2-Ab1-R**	CGGTTGTAGATGTATCTGGTCAC
**Gzmb-F**	AATGTGAAGCCAGGAGATGTG
**Gzmb-R**	CTCAACCTCTTGTAGCGTGTT
**H2-Eb1-F**	CTTGTGCGGCGGAGAGTTGA
**H2-Eb1-R**	CAGAGCAGACCAGGAGGTTGTG
**Ifi204-F**	GCAAGGCGGCTAAGGAACCA
**Ifi204-R**	TGAGTGGAGAACAGCACCTCTG
**Ifi211-F**	GCAAGGCGGCTAAGGAACCA
**Ifi211-R**	TGAGTGGAGAACAGCACCTCTG
**Zbp1-F**	GTTGACTTGAGCACAGGAGACA
**Zbp1-R**	TCTTGGGCACTTGGCATTTCTT
**Psmb9-F**	GCGGGAACAGCAGTGGTGAA
**Psmb9-R**	TTGGGCATCAGCAGCGGAAC
**Cd3d-F**	TGGAACGGTGGAAGGATGGT
**Cd3d-R**	ACAGTTCTGGCACATTCGGTAA
**Ifng-F**	CAAGTGGCATAGATGTGGAAGA
**Ifng-R**	AGGTGTGATTCAATGACGCTTA
**Cd40-F**	CTGGTCATTCCTGTCGTGATGG
**Cd40-R**	CACTGGAGCAGCGGTGTTATG
**β actin-F**	GTGACGTTGACATCCGTAAAGA
**β actin-R**	GCCGGACTCATCGTACTCC

Table S2. List of antibodies used for flow cytometry in this study.

List of antibodies used for flow cytometry			
**Target**	**Label**	**Provider**	**Catalog**
Anti-mouse CD3	PE	Biolegend	100206
Anti-mouse CD4	FITC	Biolegend	100406
Anti-mouse CD8a	PerCP	Biolegend	100732
Anti-mouse CD80	FITC	Biolegend	104706
Anti-mouse CD86	APC	Biolegend	105011
Anti-mouse Gr-1	APC	Biolegend	108412
Anti-mouse CD11c	PE	Biolegend	117308
Anti-mouse CD206	PerCP/Cyanine5.5	Biolegend	141716
Anti-mouse CD11b	PE	Biolegend	101208
Anti-mouse F4/80	APC	Biolegend	123116
Anti-mouse CD80	Pacific Blue™	Biolegend	104724
Anti-mouse IFN-γ	Alexa fluor700	Biolegend	505824
Anti-mouse IFN-γ	PE	Biolegend	505808
Anti-mouse IL-4	APC	Biolegend	504106
Anti-mouse CD45	APC	Biolegend	147708
Anti-mouse CD19	FITC	Biolegend	115506
Anti-mouse NK1.1	FITC	Biolegend	156508
Anti-mouse Granzyme B	PE	Biolegend	372208
Anti-mouse CD45	Pacific Blue™	Biolegend	157212
anti-mouse H-2Kb bound to SIINFEKL	APC	Biolegend	141606
Anti-mouse CD69	APC/Cyanine7	Biolegend	104525
Anti-mouse CD62L	BV510	Biolegend	104441
Anti-mouse CD44	FITC	eBioscience	AB_465045

Table S3. Open reading frame for mRNA vaccine design in this study.

Open reading frame for mRNA vaccine design
**OVA ORF**<br>ATGGAGACAGACACACTCCTGCTATGGGTACTGCTGCTCTGGGTTCCAGGTTCCACTGGT<br>GACGAGCAGAAACTCATCTCTGAAGAGGATCTGATGGGCTCCATCGGTGCAGCAAGCAT<br>GGAATTTTGTTTTGATGTATTCAAGGAGCTCAAAGTCCACCATGCCAATGAGAACATCTT<br>CTACTGCCCCATTGCCATCATGTCAGCTCTAGCCATGGTATACCTGGGTGCAAAAGACA<br>GCACCAGGACACAAATAAATAAGGTTGTTCGCTTTGATAAACTTCCAGGATTCGGAGAC<br>AGTATTGAAGCTCAGTGTGGCACATCTGTAAACGTTCACTCTTCACTTAGAGACATCCTC<br>AACCAAATCACCAAACCAAATGATGTTTATTCGTTCAGCCTTGCCAGTAGACTTTATGCT<br>GAAGAGAGATACCCAATCCTGCCAGAATACTTGCAGTGTGTGAAGGAACTGTATAGAG<br>GAGGCTTGGAACCTATCAACTTTCAAACAGCTGCAGATCAAGCCAGAGAGCTCATCAAT<br>TCCTGGGTAGAAAGTCAGACAAATGGAATTATCAGAAATGTCCTTCAGCCAAGCTCCGT<br>GGATTCTCAAACTGCAATGGTTCTGGTTAATGCCATTGTCTTCAAAGGACTGTGGGAGA<br>AAGCATTTAAGGATGAAGACACACAAGCAATGCCTTTCAGAGTGACTGAGCAAGAAAG<br>CAAACCTGTGCAGATGATGTACCAGATTGGTTTATTTAGAGTGGCATCAATGGCTTCTGA<br>GAAAATGAAGATCCTGGAGCTTCCATTTGCCAGTGGGACAATGAGCATGTTGGTGCTGT<br>TGCCTGATGAAGTCTCAGGCCTTGAGCAGCTTGAGAGTATAATCAACTTTGAAAAACTG<br>ACTGAATGGACCAGTTCTAATGTTATGGAAGAGAGGAAGATCAAAGTGTACTTACCTCG<br>CATGAAGATGGAGGAAAAATACAACCTCACATCTGTCTTAATGGCTATGGGCATTACTG<br>ACGTGTTTAGCTCTTCAGCCAATCTGTCTGGCATCTCCTCAGCAGAGAGCCTGAAGATAT<br>CTCAAGCTGTCCATGCAGCACATGCAGAAATCAATGAAGCAGGCAGAGAGGTGGTAGG<br>GTCAGCAGAGGCTGGAGTGGATGCTGCAAGCGTCTCTGAAGAATTTAGGGCTGACCATC<br>CATTCCTCTTCTGTATCAAGCACATCGCAACCAACGCCGTTCTCTTCTTTGGCAGATGTG<br>TTTCCCCTGGTGGAGGCGGTTCAGGCGGAGGTGGCTCTCCAAATAAAGGAAGTGGAACC<br>ACTTCAGGTACTACCCGTCTTCTATCTGGGCACACGTGTTTCACGTTGACAGGTTTGCTT<br>GGGACGCTAGTAACCATGGGCTTGCTGACTTAA
**OVA-mcherry ORF**<br>ATGGAGACAGACACACTCCTGCTATGGGTACTGCTGCTCTGGGTTCCAGGTTCCACTGGT<br>GACGAGCAGAAACTCATCTCTGAAGAGGATCTGATGGGCTCCATCGGTGCAGCAAGCAT<br>GGAATTTTGTTTTGATGTATTCAAGGAGCTCAAAGTCCACCATGCCAATGAGAACATCTT<br>CTACTGCCCCATTGCCATCATGTCAGCTCTAGCCATGGTATACCTGGGTGCAAAAGACA<br>GCACCAGGACACAAATAAATAAGGTTGTTCGCTTTGATAAACTTCCAGGATTCGGAGAC<br>AGTATTGAAGCTCAGTGTGGCACATCTGTAAACGTTCACTCTTCACTTAGAGACATCCTC<br>AACCAAATCACCAAACCAAATGATGTTTATTCGTTCAGCCTTGCCAGTAGACTTTATGCT<br>GAAGAGAGATACCCAATCCTGCCAGAATACTTGCAGTGTGTGAAGGAACTGTATAGAG<br>GAGGCTTGGAACCTATCAACTTTCAAACAGCTGCAGATCAAGCCAGAGAGCTCATCAAT<br>TCCTGGGTAGAAAGTCAGACAAATGGAATTATCAGAAATGTCCTTCAGCCAAGCTCCGT<br>GGATTCTCAAACTGCAATGGTTCTGGTTAATGCCATTGTCTTCAAAGGACTGTGGGAGA<br>AAGCATTTAAGGATGAAGACACACAAGCAATGCCTTTCAGAGTGACTGAGCAAGAAAG<br>CAAACCTGTGCAGATGATGTACCAGATTGGTTTATTTAGAGTGGCATCAATGGCTTCTGA<br>GAAAATGAAGATCCTGGAGCTTCCATTTGCCAGTGGGACAATGAGCATGTTGGTGCTGT<br>TGCCTGATGAAGTCTCAGGCCTTGAGCAGCTTGAGAGTATAATCAACTTTGAAAAACTG<br>ACTGAATGGACCAGTTCTAATGTTATGGAAGAGAGGAAGATCAAAGTGTACTTACCTCG<br>CATGAAGATGGAGGAAAAATACAACCTCACATCTGTCTTAATGGCTATGGGCATTACTG<br>ACGTGTTTAGCTCTTCAGCCAATCTGTCTGGCATCTCCTCAGCAGAGAGCCTGAAGATAT<br>CTCAAGCTGTCCATGCAGCACATGCAGAAATCAATGAAGCAGGCAGAGAGGTGGTAGG<br>GTCAGCAGAGGCTGGAGTGGATGCTGCAAGCGTCTCTGAAGAATTTAGGGCTGACCATC<br>CATTCCTCTTCTGTATCAAGCACATCGCAACCAACGCCGTTCTCTTCTTTGGCAGATGTG<br>TTTCCCCTATGGTGAGCAAGGGCGAGGAGGATAACATGGCCATCATCAAGGAGTTCATG<br>CGCTTCAAGGTGCACATGGAGGGCTCCGTGAACGGCCACGAGTTCGAGATCGAGGGCG<br>AGGGCGAGGGCCGCCCCTACGAGGGCACCCAGACCGCCAAGCTGAAGGTGACCAAGGG<br>TGGCCCCCTGCCCTTCGCCTGGGACATCCTGTCCCCTCAGTTCATGTACGGCTCCAAGGC<br>CTACGTGAAGCACCCCGCCGACATCCCCGACTACTTGAAGCTGTCCTTCCCCGAGGGCTT<br>CAAGTGGGAGCGCGTGATGAACTTCGAGGACGGCGGCGTGGTGACCGTGACCCAGGAC<br>TCCTCCCTGCAGGACGGCGAGTTCATCTACAAGGTGAAGCTGCGCGGCACCAACTTCCC<br>CTCCGACGGCCCCGTAATGCAGAAGAAGACCATGGGCTGGGAGGCCTCCTCCGAGCGG<br>ATGTACCCCGAGGACGGCGCCCTGAAGGGCGAGATCAAGCAGAGGCTGAAGCTGAAGG<br>ACGGCGGCCACTACGACGCTGAGGTCAAGACCACCTACAAGGCCAAGAAGCCCGTGCA<br>GCTGCCCGGCGCCTACAACGTCAACATCAAGTTGGACATCACCTCCCACAACGAGGACT<br>ACACCATCGTGGAACAGTACGAACGCGCCGAGGGCCGCCACTCCACCGGCGGCATGGA<br>CGAGCTGTACAAGGGTGGAGGCGGTTCAGGCGGAGGTGGCTCTCCAAATAAAGGAAGT<br>GGAACCACTTCAGGTACTACCCGTCTTCTATCTGGGCACACGTGTTTCACGTTGACAGGT<br>TTGCTTGGGACGCTAGTAACCATGGGCTTGCTGACTTAA
**HBsAg ORF**<br>ATGGAGACAGACACACTCCTGCTATGGGTACTGCTGCTCTGGGTTCCAGGTTCCACTGGT<br>GACGAGCAGAAACTCATCTCTGAAGAGGATCTGATGGAGAACATCACATCAGGATTCCT<br>AGGACCCCTTCTCGTGTTACAGGCGGGGTTTTTCTTGTTGACAAGAATCCTCACAATACC<br>GCAGAGTCTAGACTCGTGGTGGACTTCTCTCAATTTTCTAGGGGGAACTACCGTGTGTCT<br>TGGCCAAAATTCGCAGTCCCCAACCTCCAATCACTCACCAACCTCTTGTCCTCCAACTTG

TCCTGGTTATCGCTGGATGTGTCTGCGGCGTTTTATCATCTTCCTCTTCATCCTGCTGCTA
TGCCTCATCTTCTTGTTGGTTCTTCTGGACTATCAAGGTATGTTGCCCGTTTGTCCTCTAA
TTCCAGGATCCTCAACAACCAGCACGGGACCATGCCGGACCTGCATGACTACTGCTCAA
GGAACCTCTATGTATCCCTCCTGTTGCTGTACCAAACCTTCGGACGGAAATTGCACCTGT
ATTCCCATCCCATCATCCTGGGCTTTCGGAAAATTCCTATGGGAGTGGGCCTCAGCCCGT
TTCTCCTGGCTCAGTTTACTAGTGCCATTTGTTCAGTGGTTCGTAGGGCTTTCCCCCACTG
TTTGGCTTTCAGTTATATGGATGATGTGGTATTGGGGGCCAAGTCTGTACAGCATCTTGA
GTCCCTTTTTACCGCTGTTACCAATTTTCTTTTGTCTTTGGGTATACATTGGTGGAGGCGG
TTCAGGCGGAGGTGGCTCTCCAAATAAAGGAAGTGGAACCACTTCAGGTACTACCCGTC
TTCTATCTGGGCACACGTGTTTCACGTTGACAGGTTTGCTTGGGACGCTAGTAACCATGG
GCTTGCTGACTTAA

SRBD ORF

ATGGAGACAGACACACTCCTGCTATGGGTACTGCTGCTCTGGGTTCCAGGTTCCACTGGT
GACGAGCAGAAACTCATCTCTGAAGAGGATCTGAGAGTCCAACCAACAGAATCTATTGT
TAGATTTCCTAATATTACAAACTTGTGCCCTTTTGGTGAAGTTTTTAACGCCACCAGATT
TGCATCTGTTTATGCTTGGAACAGGAAGAGAATCAGCAACTGTGTTGCTGATTATTCTGT
CCTATATAATTCCGCATCATTTTCCACTTTTAAGTGTTATGGAGTGTCTCCTACTAAATTA
AATGATCTCTGCTTTACTAATGTCTATGCAGATTCATTTGTAATTAGAGGTGATGAAGTC
AGACAAATCGCTCCAGGGCAAACTGGAAAGATTGCTGATTATAATTATAAATTACCAGA
TGATTTTACAGGCTGCGTTATAGCTTGGAATTCTAACAATCTTGATTCTAAGGTTGGTGG
TAATTATAATTACCTGTATAGATTGTTTAGGAAGTCTAATCTCAAACCTTTTGAGAGAGA
TATTTCAACTGAAATCTATCAGGCCGGTAGCACACCTTGTAATGGTGTTGAAGGTTTTAA
TTGTTACTTTCCTTTACAATCATATGGTTTCCAACCCACTAATGGTGTTGGTTACCAACCA
TACAGAGTAGTAGTACTTTCTTTTGAACTTCTACATGCACCAGCAACTGTTTGTGGACCT
AAAAAGTCTACTAATTTGGTTAAAAACAAATGTGTCAATTTCGGTGGAGGCGGTTCAGG
CGGAGGTGGCTCTCCAAATAAAGGAAGTGGAACCACTTCAGGTACTACCCGTCTTCTAT
CTGGGCACACGTGTTTCACGTTGACAGGTTTGCTTGGGACGCTAGTAACCATGGGCTTGC
TGACTTAA

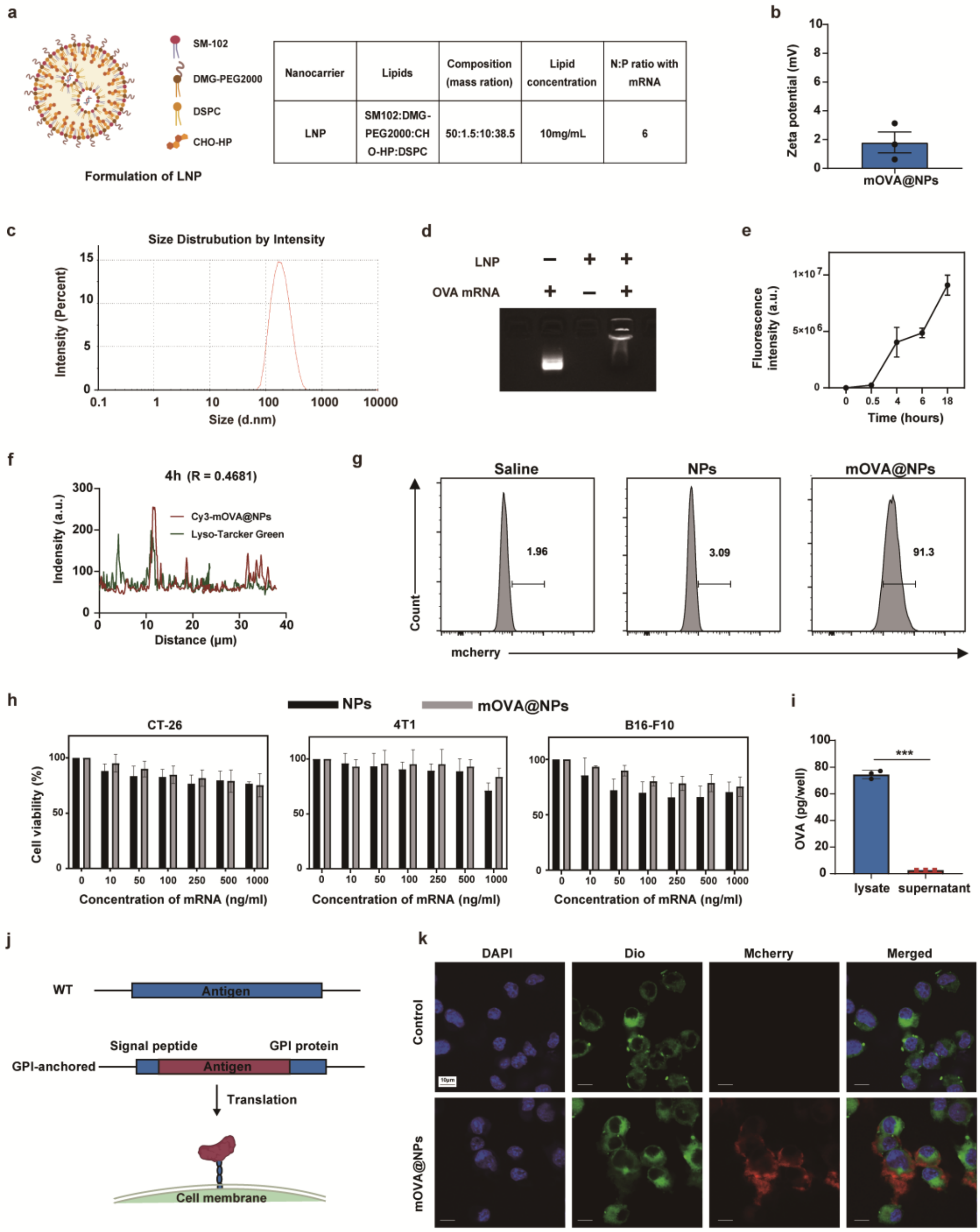

Figure S1. Characterization of mRNA vaccine using model antigen ovalbumin.

a) Formulation of LNP nanoparticles by Moderna: SM102, 1-octylnonyl 8-[(2-hydroxyethyl)[6-oxo-6-(undecyloxy)hexyl]amino]octanoate; DMG-PEG2000, 1,2-dimyristoyl-rac-glycero-3-methoxypolyethylene glycol-2000; CHO-HP, Cholest-5-en-3β-ol; DSPC, 1,2-dioctadecanoyl-sn-glycero-3-phophocholine. **b)** Zeta potential of mOVA@NPs. **c)** Size distribution of mOVA@NPs. **d)** RNA gel imaging for mRNA loading by NPs. **e)** Quantification of fluorescence intensity of Cy3-mOVA@NPs at 0, 0.5, 4, 6 and 18 hours. **f)** Quantification of co-localization at 4 hours using Pearson-coefficient. **g)** Flow cytometry analysis of the percentage of mcherry-tagged OVA (red) expression in B16-F10 cells treated with NPs and

mOVA@NPs for 48 hours. **h)** Cell viability of CT-26, 4T1 and B16-F10 tumor cells after incubation with NPs and mOVA@NPs for 24 hours. **i)** OVA expression in B16-F10 cell lysates and supernatants analyzed by ELISA after mOVA@NPs transfection for 24 hours. **j)** Design of membrane-anchored mRNA encoding for antigen for intratumoral injection. **k)** Orientation of anchored antigens in membranes by CLSM imaging. Scale bar, 10 μm. All data are expressed as means ± SDs. Statistical significance was calculated by Student's t-test, two-tailed. *P < 0.05; **P < 0.01; ***P < 0.001; NS, not significant.

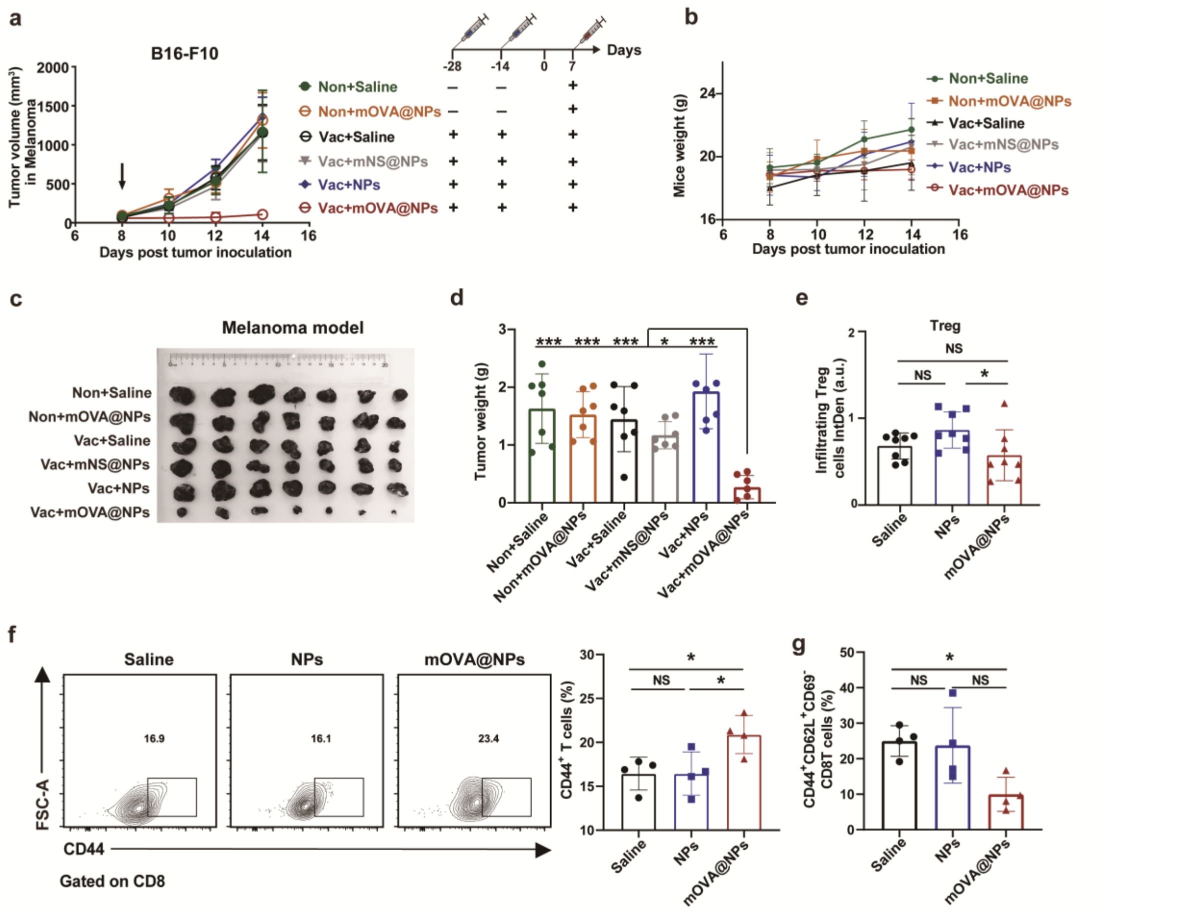

Figure S2. mOVA@NPs elicit strong anti-tumor effects in melanoma cancer models.
a) The average tumor volume curves for mice in melanoma cancer model (n = 7). **b)** The mice weight in melanoma cancer model (n = 7). **c, d)** The tumor image (c) and tumor weight (d) in melanoma cancer model (n = 7). **e)** Quantification of signal intensity of FOXP3 expression in Fig. 3E in melanoma cancer model. **f)** Flow cytometry analysis of the level of $CD44^+CD8^+$ T cells within tumor (n = 4). **g)** Quantification of $CD62L^-CD69^+$ cells gated on $CD44^+CD8^+$ T cells (n = 4), as shown in Figure 3f. All data are expressed as means ± SDs. Statistical significance was calculated by a one-way ANOVA with a Tukey's multiple comparisons test. *P < 0.05; **P < 0.01; ***P < 0.001; NS, not significant.

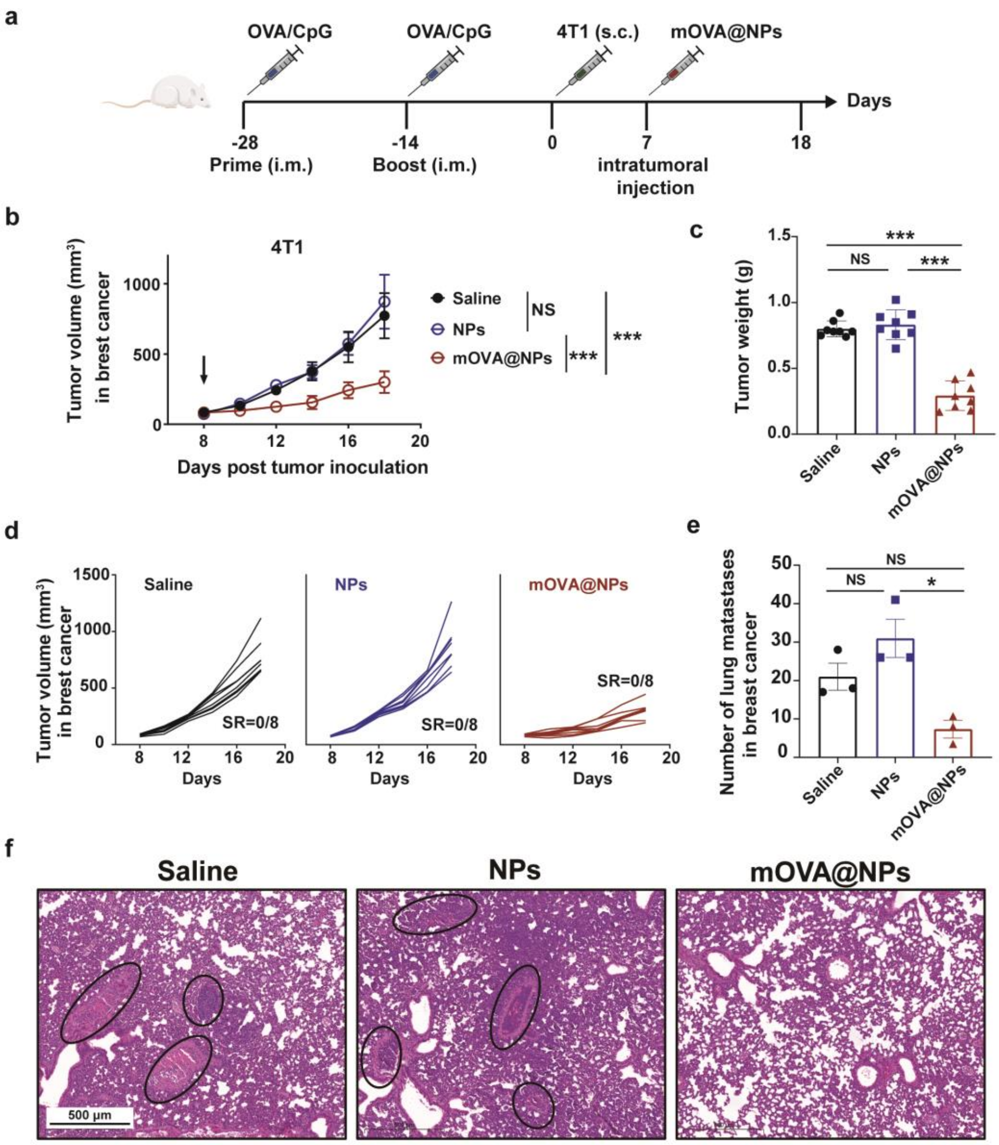

Figure S3. Anti-tumor effects of mice treated with saline, control NPs and mOVA@NPs in 4T1 cancer model.

a) Experimental timeframe for OVA vaccination and intratumoral mOVA@NPs administration in 4T1 tumor-bearing mice. **b)** The average tumor volume curves for mice treated in the breast cancer model (n = 8). **c)** The tumor weight for mice treated in the breast cancer model (n = 8). **d)** The individual tumor volume curves for mice treated in the breast cancer model (n = 8). **e, f)** Quantification and representative image of lung metastasis in the breast cancer model (n = 3). Black boxes represent lung metastases. Scale bar, 500 μm. All data are expressed as means ± SDs. Statistical significance was calculated by a one-way ANOVA with a Tukey's multiple comparisons test. *P < 0.05; **P < 0.01; ***P < 0.001; NS, not significant.

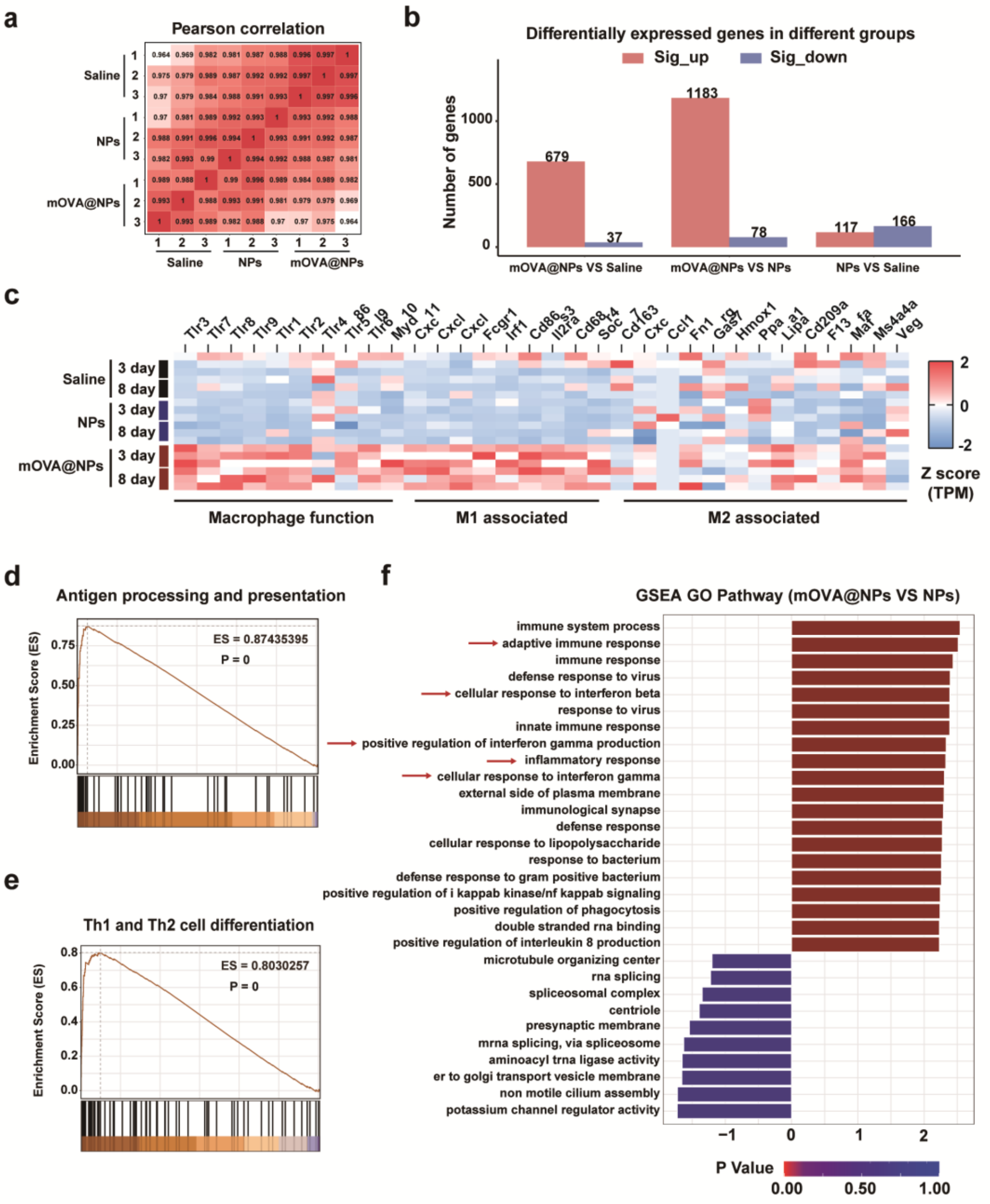

Figure S4. Transcriptomic RNA-seq identified differentially-expressed immune-relevant genes in tumors after mRNA vaccination.

a) Pearson correlation between different samples. **b)** Number of genes up-regulated and down-regulated among saline, NPs, and mOVA@NPs groups. **c)** Heatmap of gene expression associated with macrophages in tumors after treatment with saline, NPs and mOVA@NPs. **d, e)** Enrichment scores of pathways (antigen processing and presentation, th1 and th2 cell differentiation) from tumors treated with NPs and mOVA@NPs. **f)** GSEAs demonstrating the top 30 enriched GO pathways between mOVA@NPs groups and NPs groups.

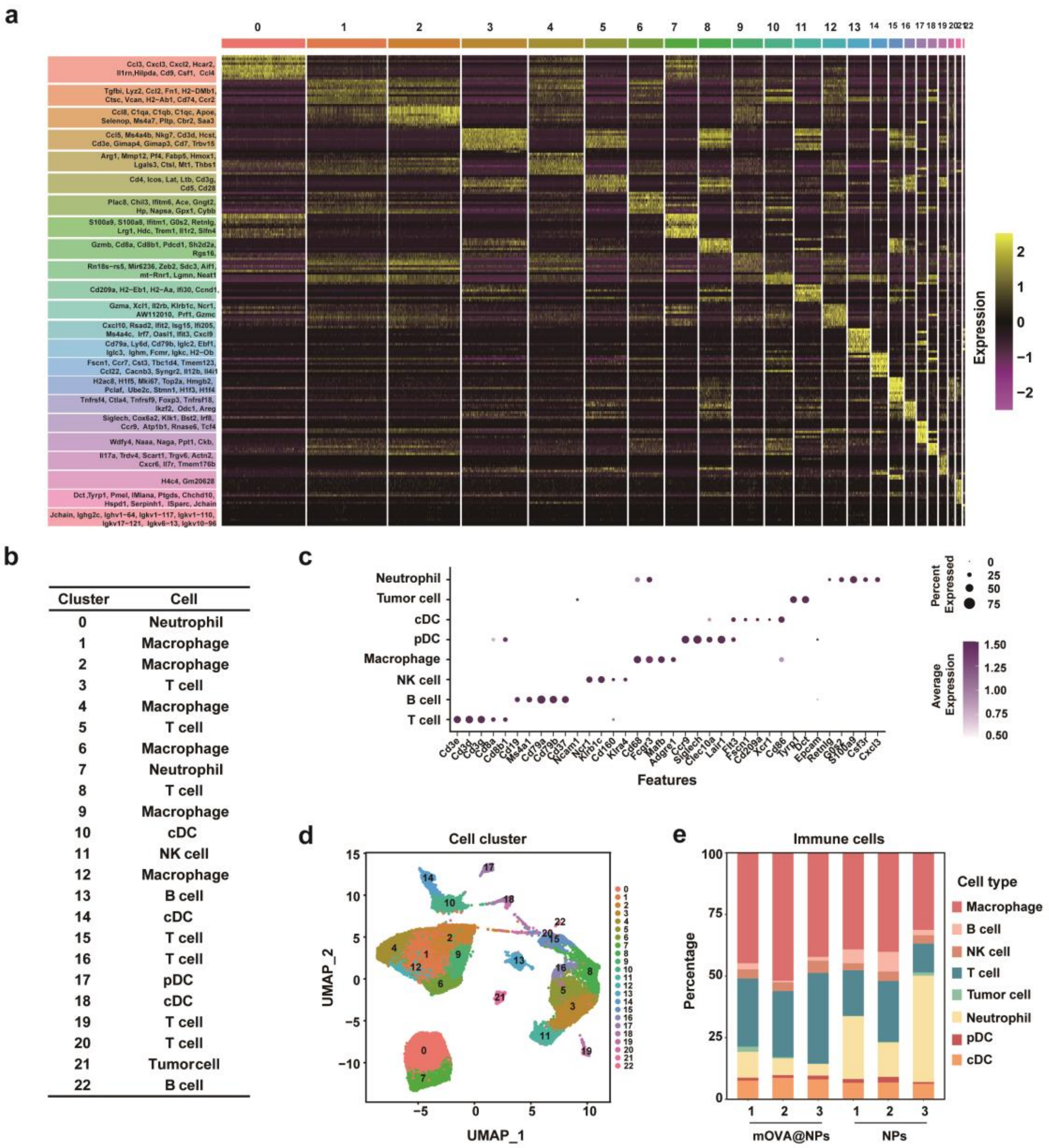

Figure S5. scRNA-seq analyses unveil major immune populations in the TME after mRNA vaccination.

a) Sequenced cells were divided into 23 clusters, and representative genes in each cluster are shown on the left. **b)** 23 clusters were assigned to 8 types of cells. **c)** Marker gene expression for each cell type, with dot color and size representing the average scaled expression value and percentage of marker gene expression, respectively. **d)** The UMAP plot of immune cells from the NPs-treated tumors and mOVA@NPs treated tumors (n = 3), with each cell color-coded by cell cluster. **e)** Histogram of the proportion of each type of immune cells in biological samples.

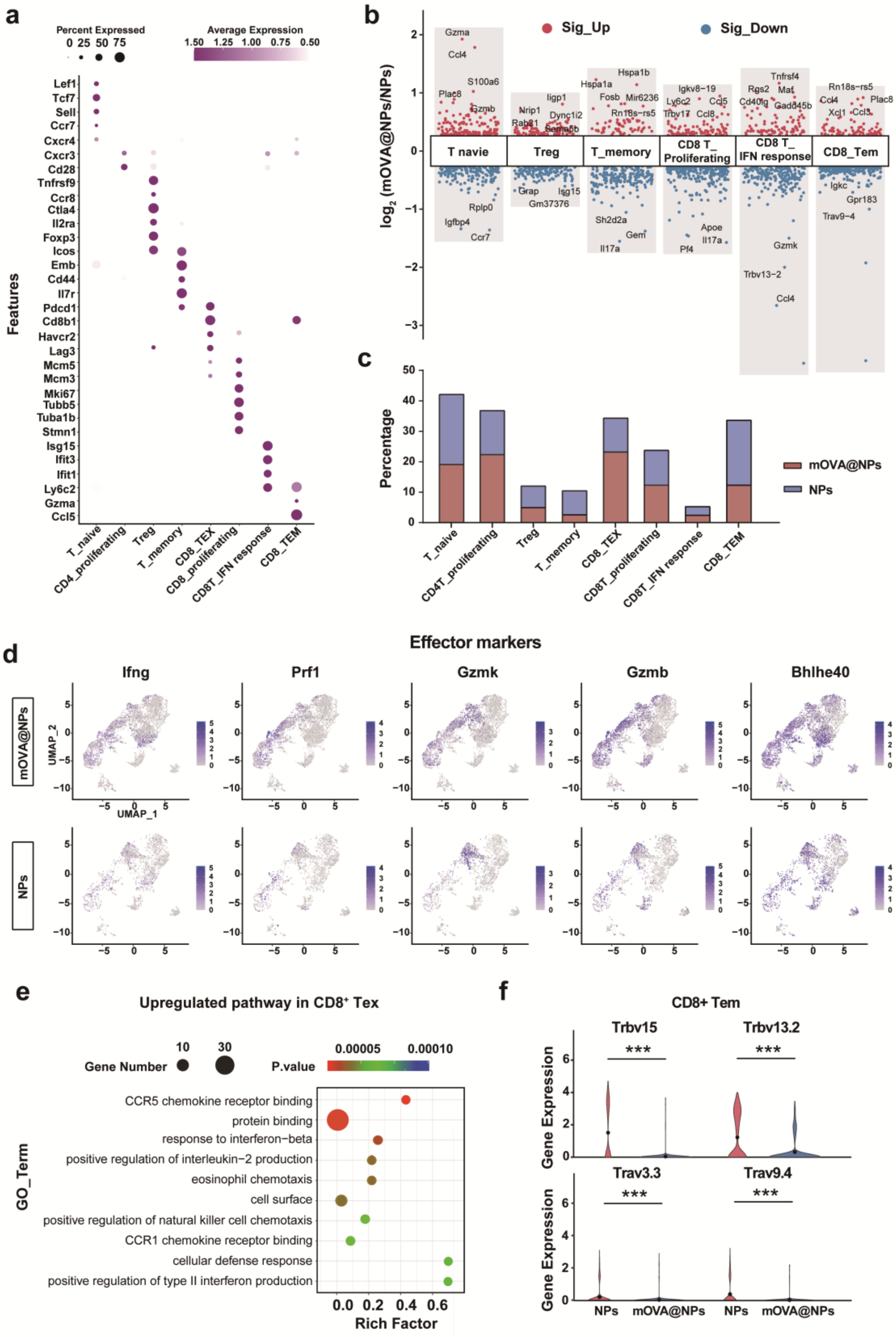

Figure S6. scRNA-seq analyses of T cell subpopulations after mRNA vaccination.

a) Dot-plot was used to show the identity of 8 subtypes of T cells. **b)** Differential gene expression analysis showing up-regulated and down-regulated genes across all 8 T-cell subpopulations. **c)** Histogram depicting the percentage of all 8 T-cell subpopulations. **d)** UMAP

plot of T-cell subclusters in the mOVA@NPs group and NPs group, color-coded by the abundance of marker genes. **e)** GO enrichment analysis for exhausted CD8^{+} T cells. Genes up-regulated in the mOVA@NPs group were collected for enrichment analysis. **f)** Violin plots of the expression levels of Tra and Trb genes in the CD8^{+} Tem subset of mOVA@NPs-treated tumors compared to the control NPs group. Two-tailed unpaired Student's t-test. *P < 0.05; **P < 0.01; ***P < 0.001; NS, not significant.

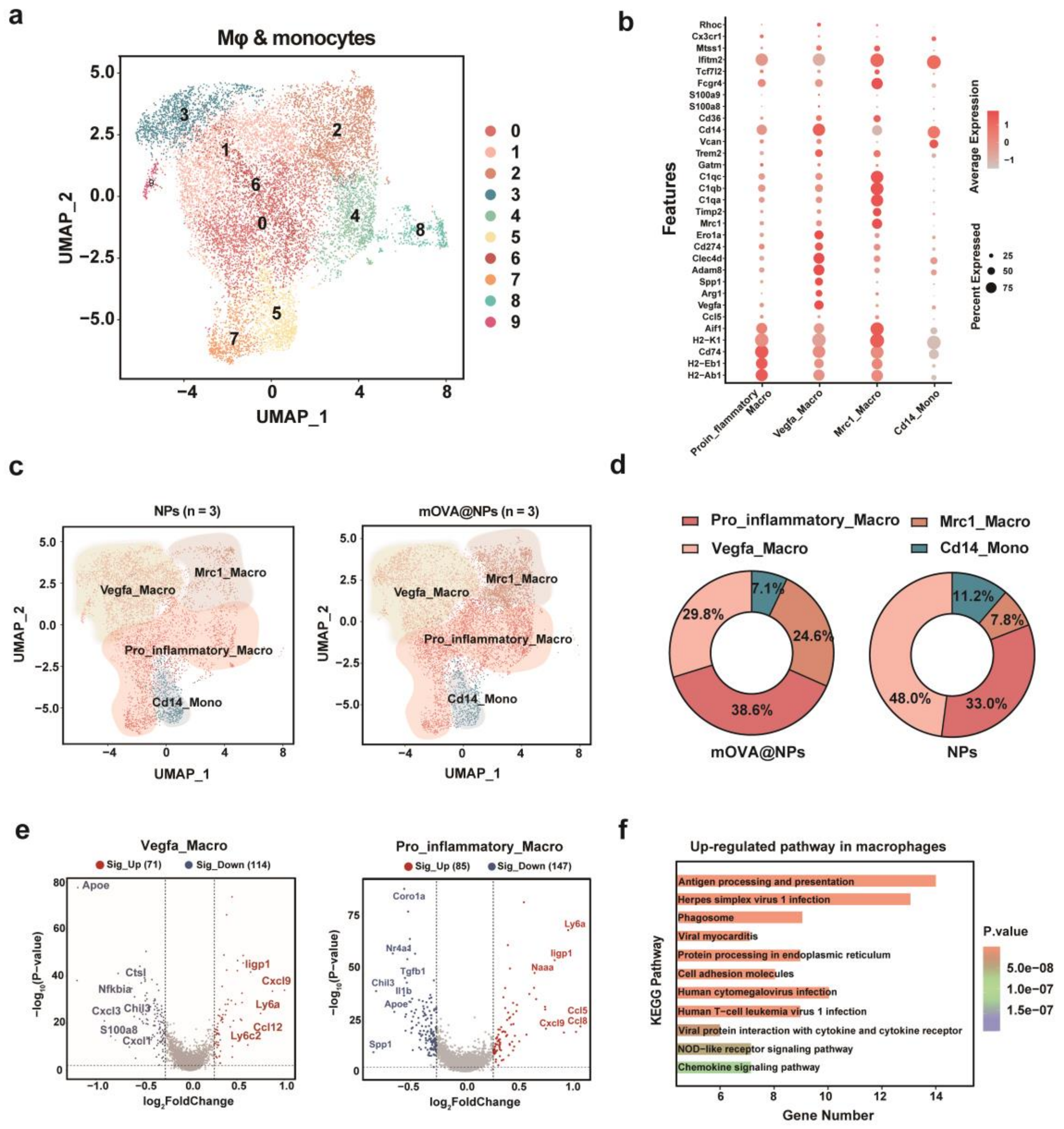

Figure S7. scRNA-seq analyses of macrophage subpopulations after mRNA vaccination. **a)** The UMAP plot of macrophages. **b)** Dot-plot was used to show the identity of 4 subtypes of macrophages. **c)** The UMAP plot of macrophages isolated from melanoma tissues in the mOVA@NPs and NPs groups, with each cell color-coded by cell cluster. **d)** Bar plot showing the percentage of each macrophage subpopulation in the mOVA@NPs and NPs groups. **e)** Volcano plots of differentially expressed genes in Vegfa macrophages and Pro-inflammatory macrophages from the mOVA@NP and NPs groups. **f)** KEGG pathway enrichment analysis for macrophages. Genes upregulated in the mOVA@NPs group were collected for enrichment analysis.

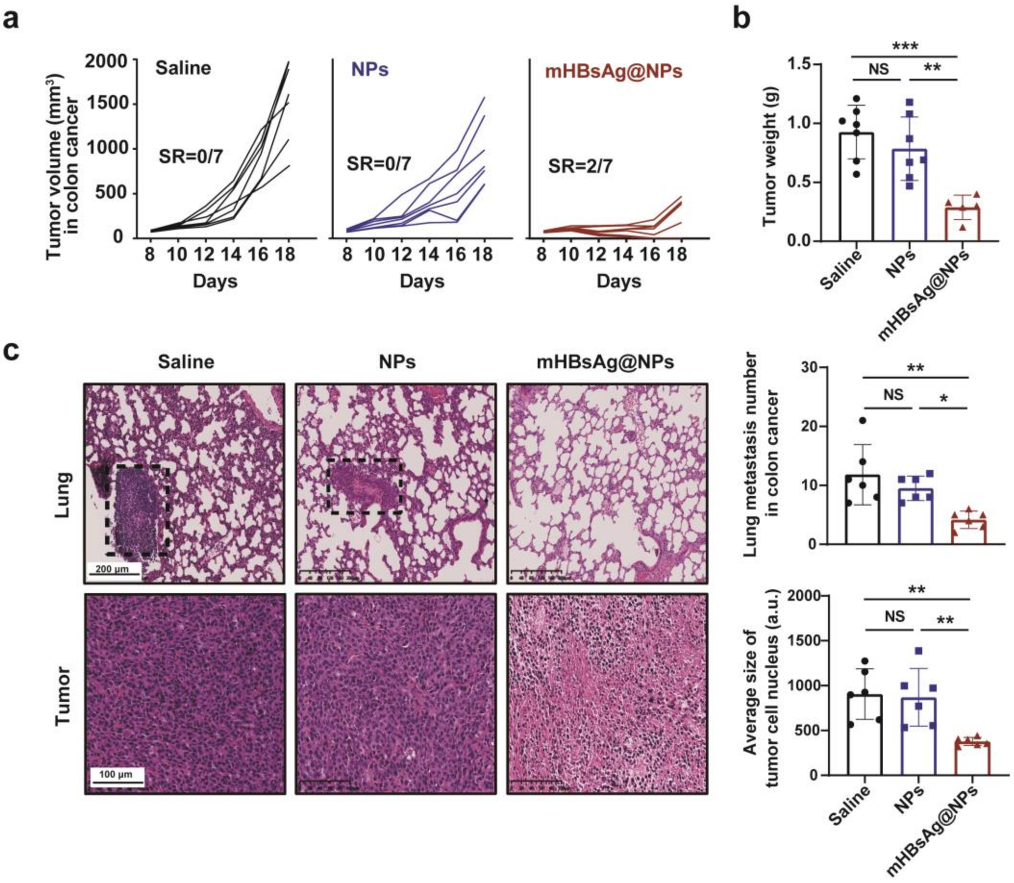

Figure S8. Anti-tumor effects of mice treated with saline, control NPs and mHBsAg@NPs in CT-26 cancer model.

a) The individual tumor volume curves for mice treated in colon tumor model (n = 7). **b)** The tumor weight for mice treated by saline, NPs and mHBsAg@NPs in colon cancer model (n = 7). **c)** Representative H&E staining and quantification of lung cancer metastasis and average size of tumor cells nucleus treated by saline, NPs and mHBsAg@NPs in colon cancer model (n = 6). Scale bars of upper represent 200 µm and lower represent 100 µm. Statistical significance was calculated by a one-way ANOVA with a Tukey's multiple comparisons test. *P < 0.05; **P < 0.01; ***P < 0.001; NS, not significant.

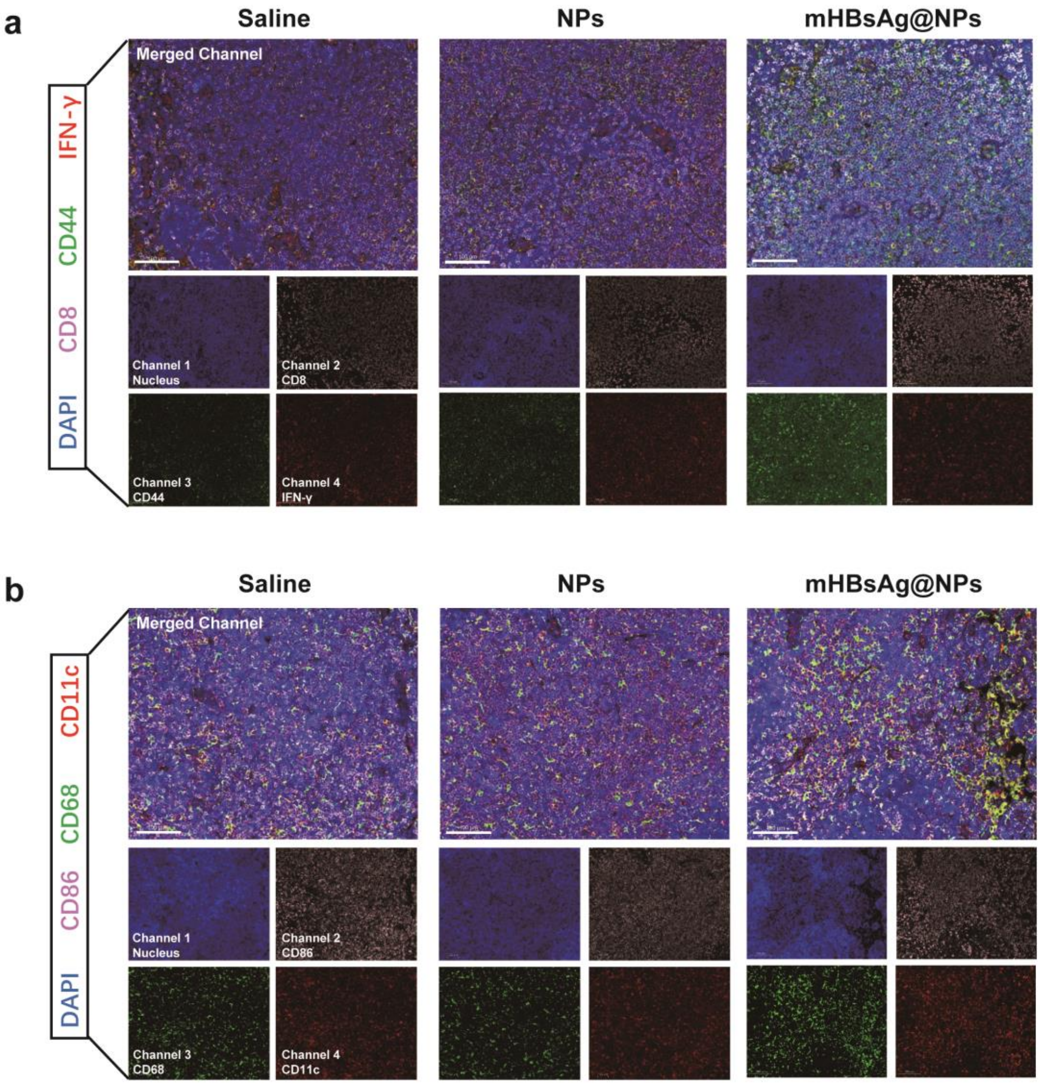

Figure S9. Representative immunofluorescence staining of lymph nodes from mice treated with saline, control NPs, and mHBsAg@NPs in CT-26 cancer model. The lymph nodes were collected for analyzing the activation (IFN-γ^{+}, red) of memory CD8 T cells (CD44^{+}, green; CD8^{+}, pink) **(a)**, as well as and the activation (CD86^{+}, pink) of DCs (CD11c^{+}, red) and macrophages (CD68^{+}, green) **(b)** by immunofluorescence staining of histological sections. Scale bars, 100 μm.

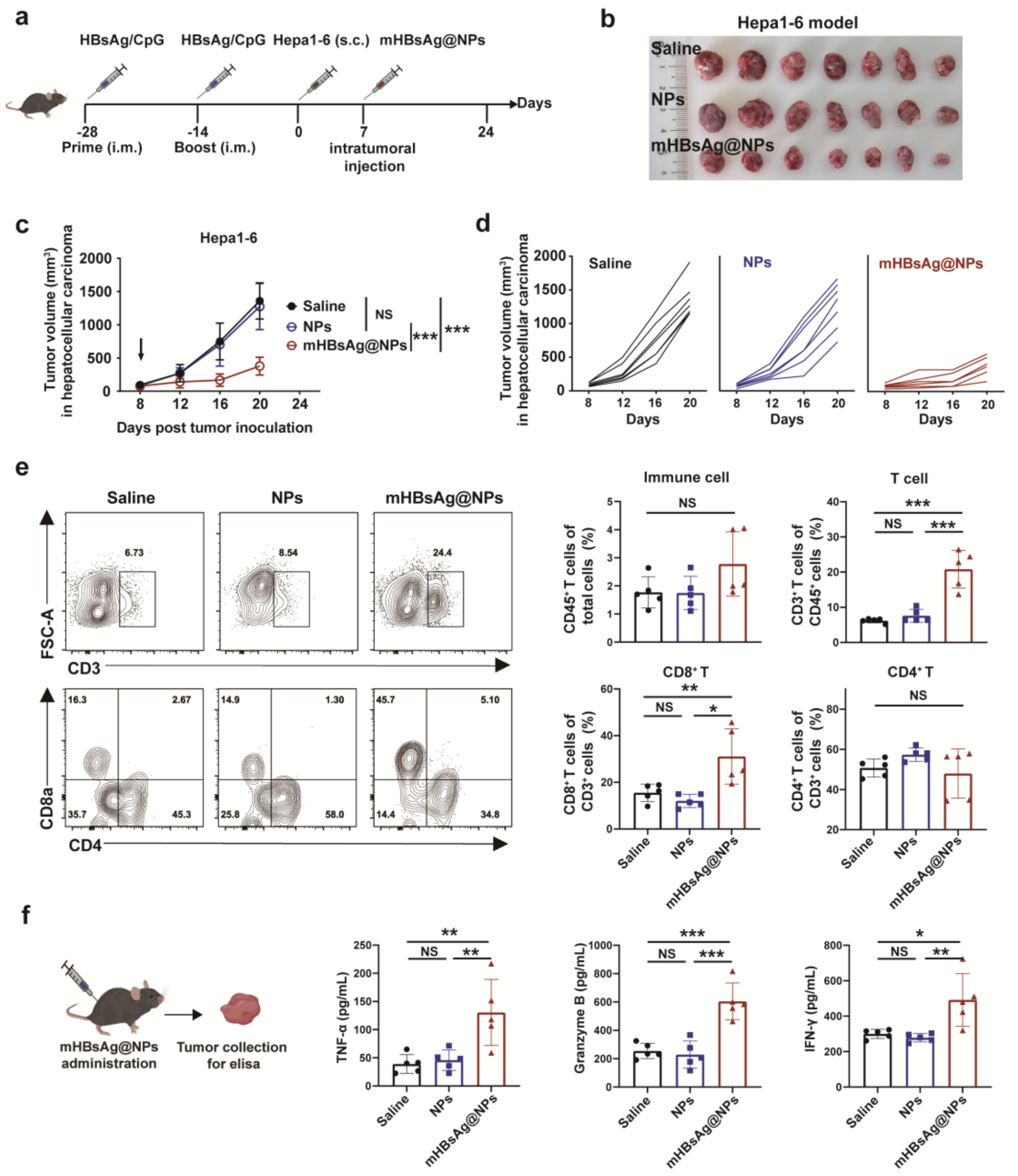

Figure S10. Anti-tumor effects of mice treated with saline, control NPs and mHBsAg@NPs in Hepa1-6 cancer model.

a) Experimental timeframe for HBsAg vaccination and mHBsAg@NPs administration in Hepa1-6 tumor-bearing mice. **b)** The tumor images of mice treated in the hepatocellular carcinoma cancer model (n = 7). **c, d)** The average and individual tumor volume curves for mice treated in the hepatocellular carcinoma cancer model (n = 7). **e)** Flow cytometry analysis of immune cells, T cells, $CD3^+/CD8^+$ T cells, $CD3^+/CD4^+$ T within tumors (n = 5). **f)** ELISA analysis of cytokines in tumor tissues (n = 5), including TNF-α, Granzyme B, and IFN-γ. All data are expressed as means ± SDs. Statistical significance was calculated by a one-way ANOVA with a Tukey's multiple comparisons test. *P < 0.05; **P < 0.01; ***P < 0.001; NS, not significant.

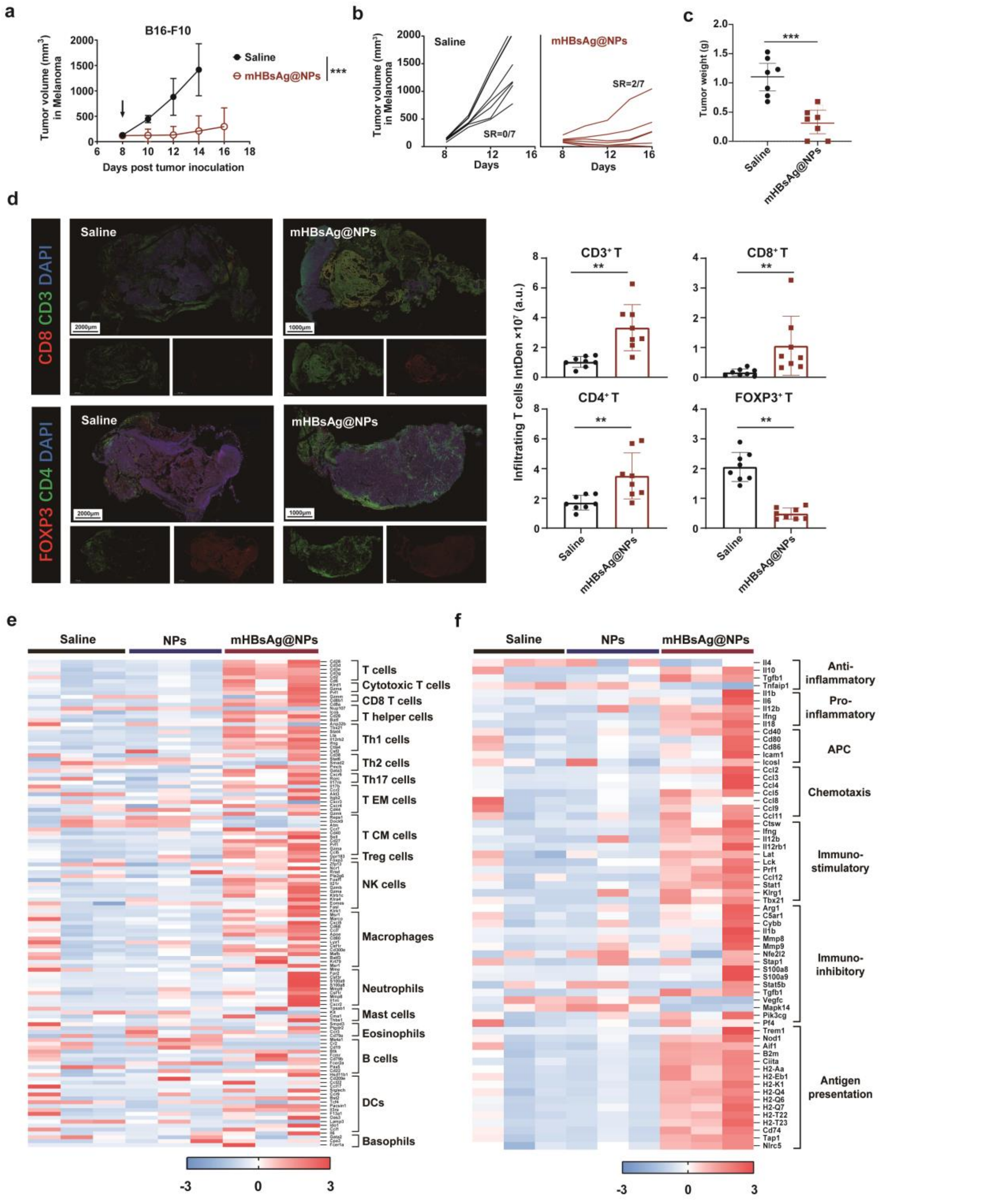

Figure S11. Anti-tumor effects of mice treated with saline, control NPs and mHBsAg@NPs in B16-F10 cancer model.

a, b) The average and individual tumor volume curves for mice treated in the melanoma model (n = 7). **c)** Tumor weight for mice treated in the melanoma model (n = 7). **d)** Representative images and quantification of the immunostaining for CD3, CD8, CD4, and FOXP3 performed in melanoma model. Scale bars represent 2,000 µm and 1,000 µm, respectively. **e)** Heatmap of the expression of genes associated with immune cell types from tumor tissues treated with saline,

NPs and mHBsAg@NPs in melanoma model. **f)** Heatmap of anti-inflammatory, proinflammatory, APC, chemotaxis family, and immunomodulation gene expression from tumor tissues with treatment of saline, NPs and mHBsAg@NPs in melanoma model. All data are expressed as means ± SDs. Statistical significance was calculated by Student's t-test, two-tailed. *P < 0.05; **P < 0.01; ***P < 0.001; NS, not significant.

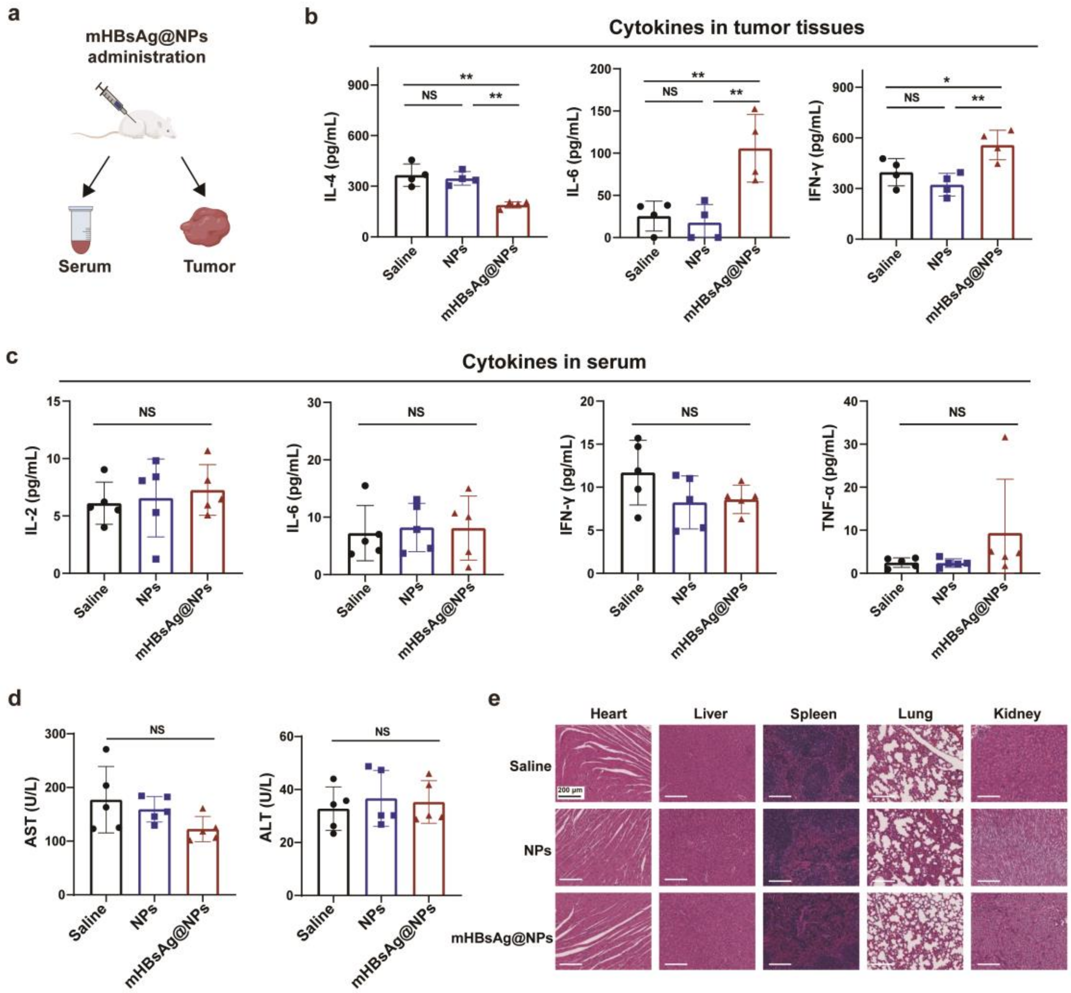

Figure S12. Biosafety of mice treated with saline, NPs and mHBsAg@NPs in 4T1 cancer model.

a) Schematic of collecting serum and tumor tissue for cytokine measuring. **b)** ELISA analysis of cytokine in tumor tissue at day 16 from mice (n = 4), including IL-4, IL-6, IFN-γ. **c)** ELISA analysis of cytokine in the serum at day 16 from mice (n = 5), including IL-2, IL-6, IFN-γ, TNF-α. **d)** AST levels and ALT levels of 4T1-bearing mice after intratumoral injection of saline, NPs and mHBsAg@NPs respectively. AST, Aspartate transaminase. ALT alanine aminotransferase. **e)** The major organs (heart, liver, spleen, lung and kidney) were collected at day 16 and analyzed by H&E staining to evaluate the biosafety. Scale bar, 200 μm. All data are expressed as means ± SDs. Statistical significance was calculated by a one-way ANOVA with a Tukey's multiple comparisons test. *P < 0.05; **P < 0.01; ***P < 0.001; NS, not significant.

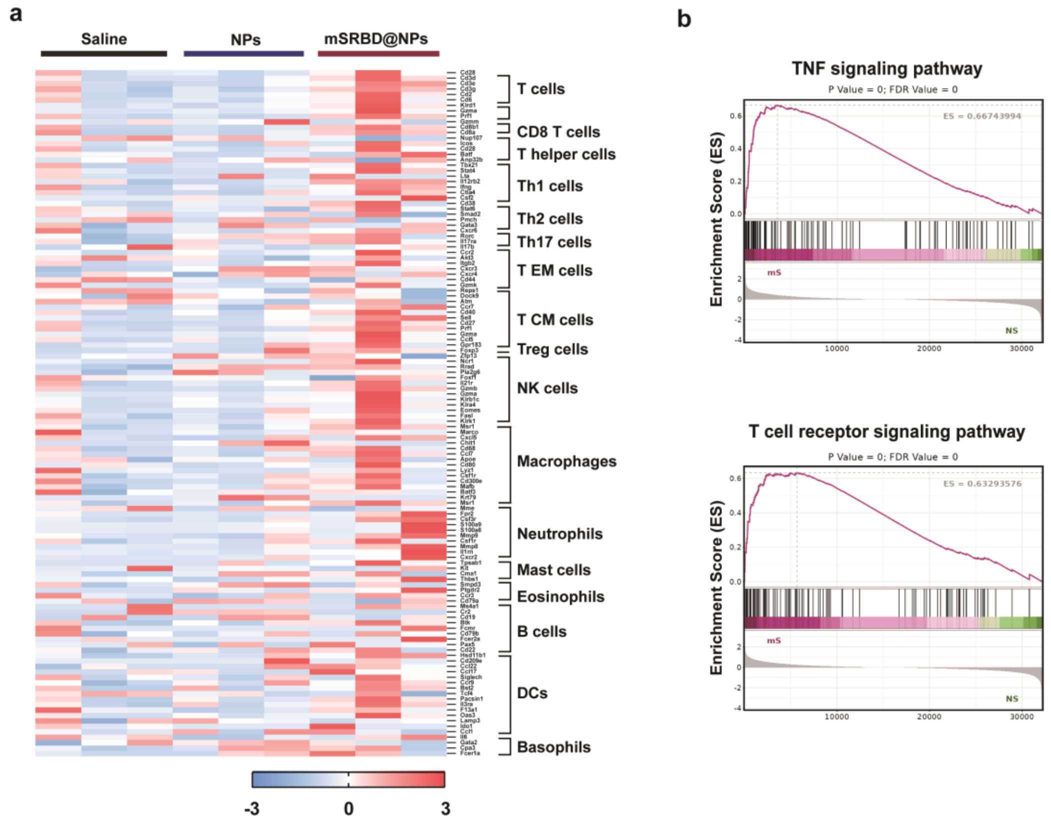

Figure S13. TME markers for tumors treated with saline, NPs, mSRBD@NPs in melanoma model.

a) Heatmap of expression of genes associated with immune cell types from tumor tissues treated with saline, NPs and mSRBD@NPs in melanoma model. **b)** Enrichment scores of the indicated pathways from tumor tissues treated with NPs and mSRBD@NPs.

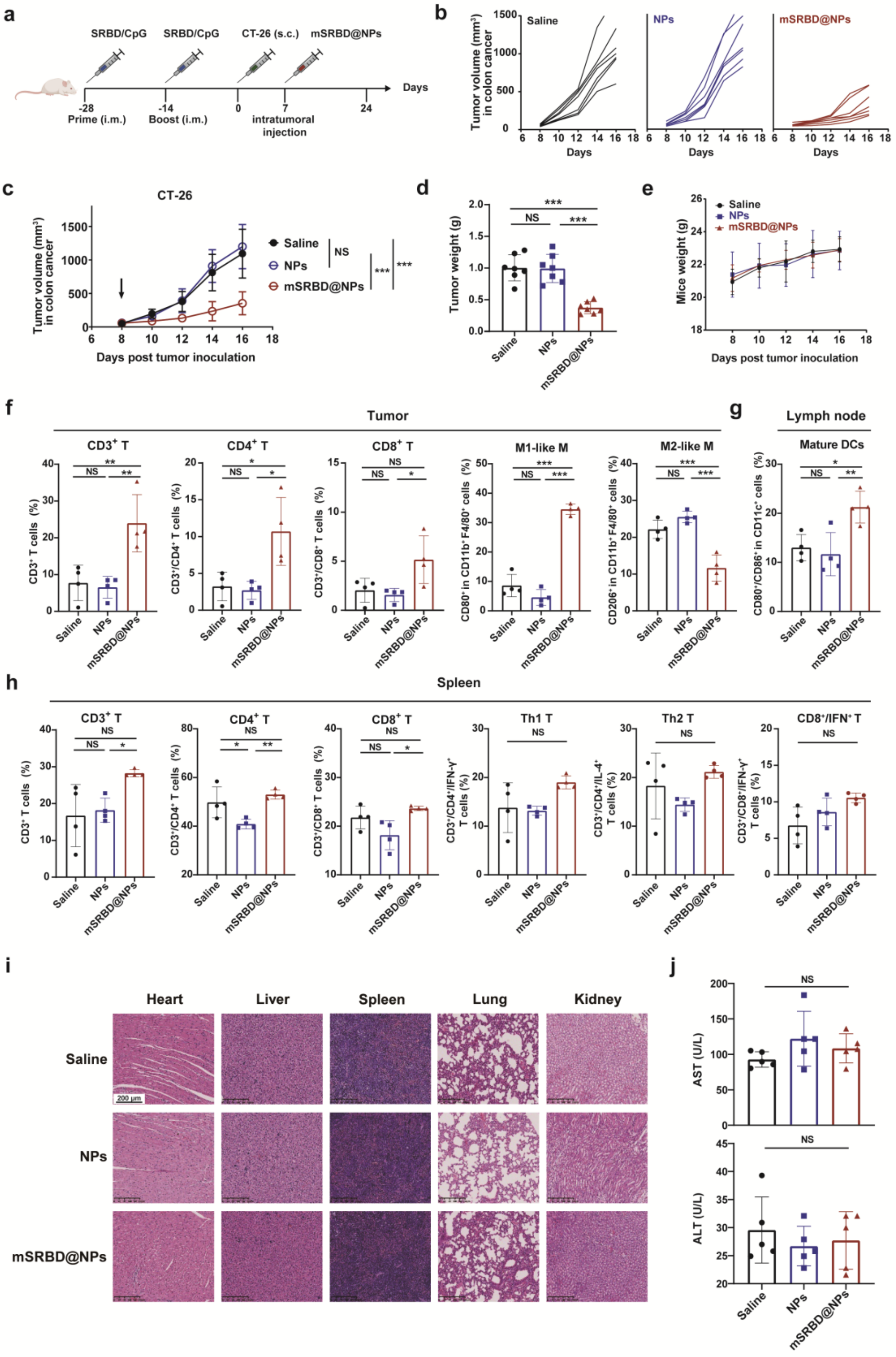

Figure S14. Anti-tumor responses of mice treated with saline, NPs and mSRBD@NPs in CT-26 cancer model.

a) Experimental timeframe for vaccination and intratumoral injection of mSRBD@NPs in CT-26 tumor-bearing mice. **b, c)** The individual and average tumor volume curves for mice treated

in the colon cancer model (n = 7). **d)** The tumor weight of tumor treated with saline, NPs and mSRBD@NPs (n = 7). **e)** The mice weight in the colon cancer model (n = 7). **f)** Flow cytometry analysis of the level of $CD3^+$ T cells, $CD3^+/CD4^+$ T cells, $CD3^+/CD8^+$ T cells, M1-like macrophages, M2-like macrophages in tumor tissues in CT-26 model (n = 4). **g)** Flow cytometry analysis of the levels of $CD11c^+/CD80^+/CD86^+$ matured DC cells in tumor draining lymph nodes in CT-26 cancer model (n = 4). **h)** Flow cytometry analysis of the levels of $CD3^+$, $CD3^+/CD4^+$, $CD3^+/CD8^+$, $CD3^+/CD4^+/IFN\text{-}\gamma^+$, $CD3^+/CD4^+/IL\text{-}4^+$, $CD3^+/CD8^+/IFN\text{-}\gamma^+$ splenocytes in CT-26 cancer model (n = 4). **i)** The major organs (heart, liver, spleen, lung and kidney) were collected and analyzed by H&E staining to evaluate the biosafety. Scale bar, 200 μm. **j)** AST levels and ALT levels of CT-26-bearing mice after intratumoral injection of saline, NPs, mSRBD@NPs respectively. All data are expressed as means ± SDs. Statistical significance was calculated by a one-way ANOVA with a Tukey's multiple comparisons test. *P < 0.05; **P < 0.01; ***P < 0.001; NS, not significant.

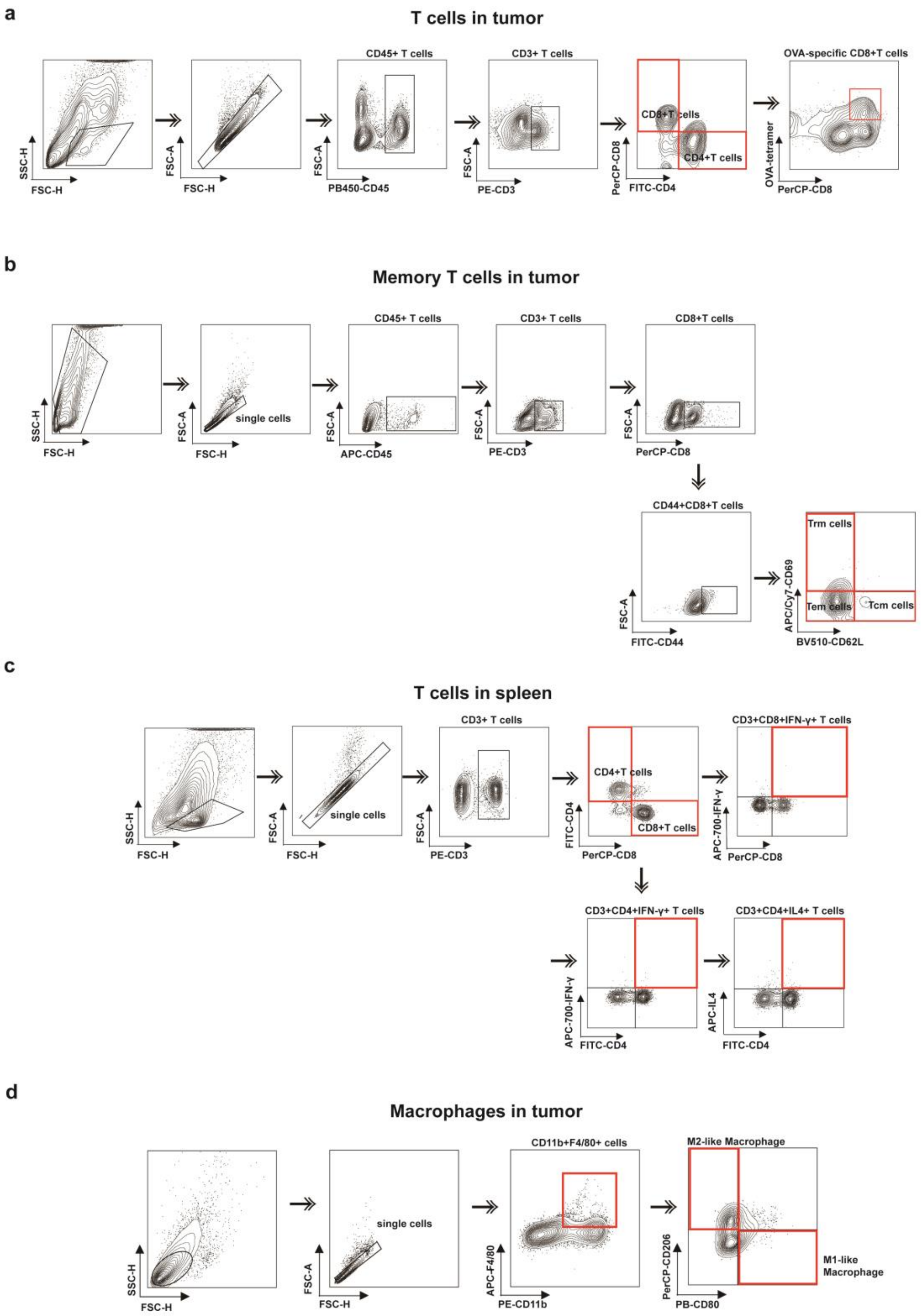

Figure S15 (a-d). Gating strategies for T cells and macrophage in tumor and spleen in this study.

Mice were euthanized and tumors or spleens were isolated after treatment. Singlet cells were selected from the cell population. For T cells, $CD3^{+}CD8^{+}$ T cells, $CD3^{+}CD4^{+}$ T cells, OVA-specific $CD8^{+}$ T cell, and memory T cells within tumor tissues were selected (a-b). Then, IFN-

$\gamma^+$$CD8^+$ T cells, $IL4^+$$CD4^+$ T cells, and IFN-$\gamma^+$$CD4^+$ T cells within spleen were analyzed (c). For macrophages, $CD11b^+$$F4/80^+$ cells were selected and $CD80^+$ macrophages (M1-like) and $CD206^+$ macrophages (M2-like) were analyzed (d).

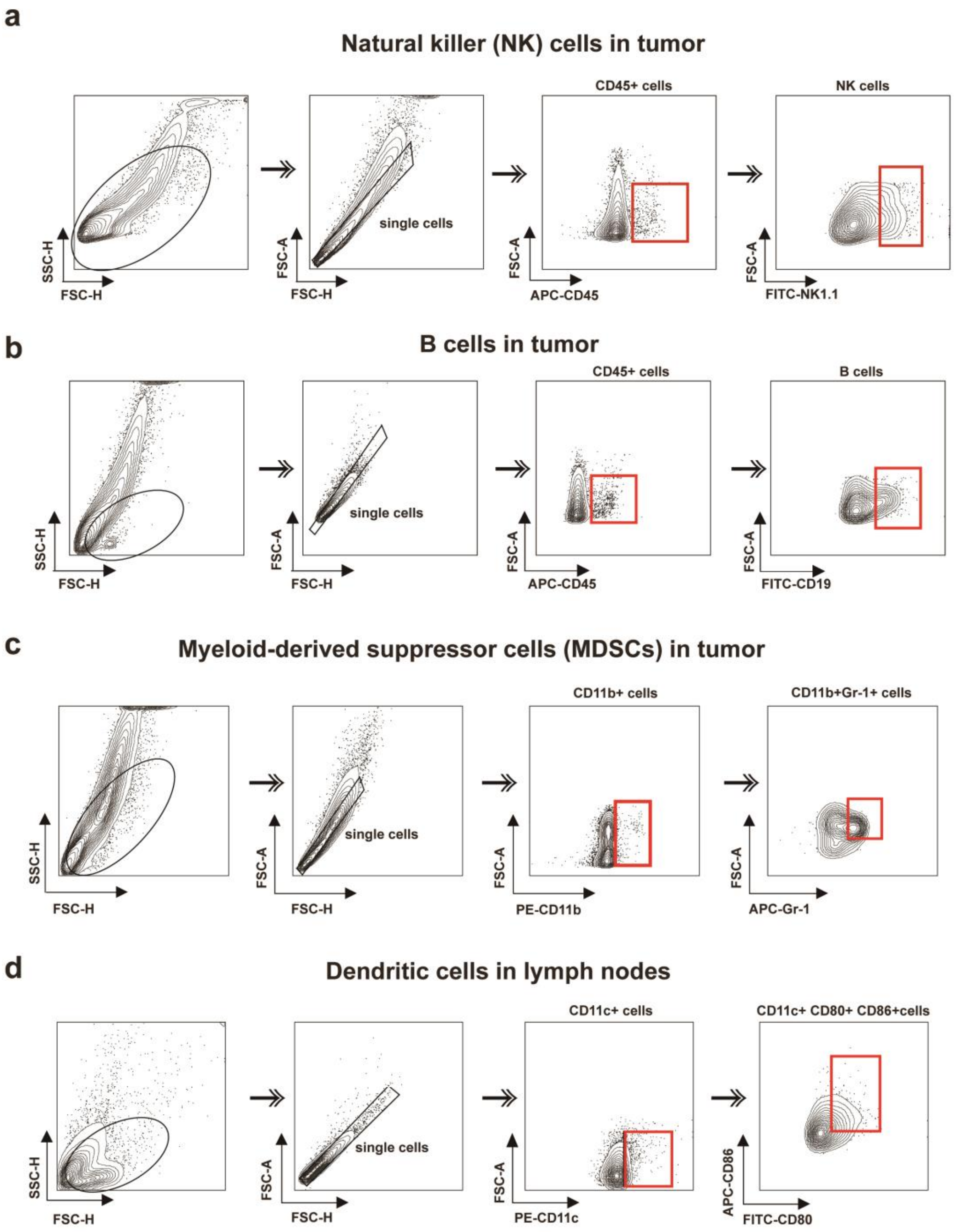

Figure S16 (a-d). Gating strategies for NK cells, B cells, myeloid-derived suppressor cells (MDSC) and dendritic cells in this study.

Mice were euthanized and tumors or lymph nodes were isolated after treatment. Singlet cells were selected from the cell population. For natural killer cells, $CD45^{+}$ $NK1.1^{+}$ cells were analyzed (a). For B cells, $CD45^{+}CD19^{+}$ cells were analyzed (b). For myeloid-derived suppressor cells, $CD11b^{+}Gr\text{-}1^{+}$ cells were analyzed (c). For mature dendritic cells in lymph nodes, $CD11c^{+}CD80^{+}$ $CD86^{+}$ cells were analyzed (d).
